# Hedgehog mediated degradation of Ihog adhesion proteins modulates cell segregation in *Drosophila* wing imaginal discs

**DOI:** 10.1038/s41467-017-01364-z

**Published:** 2017-11-02

**Authors:** Elaine Y. C. Hsia, Ya Zhang, Hai Son Tran, Agnes Lim, Ya-Hui Chou, Ganhui Lan, Philip A. Beachy, Xiaoyan Zheng

**Affiliations:** 1 Department of Anatomy and Regenerative Biology, George Washington University School of Medicine and Health Sciences, Washington, DC 20037 USA; 2 GW Cancer Center, George Washington University School of Medicine and Health Sciences, Washington, DC 20052 USA; 3 Departments of Biochemistry and Developmental Biology, Institute for Stem Cell Biology and Regenerative Medicine, Stanford University School of Medicine, Stanford, CA 94305 USA; 4 Howard Hughes Medical Institute, Stanford University School of Medicine, Stanford, CA 94305 USA; 5 Institute of Cellular and Organismic Biology, Academia Sinica, Taipei, 115 Taiwan; 6 Department of Physics, George Washington University, Washington, DC 20052 USA

## Abstract

The *Drosophila* Hedgehog receptor functions to regulate the essential downstream pathway component, Smoothened, and to limit the range of signaling by sequestering Hedgehog protein signal within imaginal disc epithelium. Hedgehog receptor function requires both Patched and Ihog activity, the latter interchangeably encoded by interference hedgehog (*ihog*) or brother of ihog (*boi*). Here we show that Patched and Ihog activity are mutually required for receptor endocytosis and degradation, triggered by Hedgehog protein binding, and causing reduced levels of Ihog/Boi proteins in a stripe of cells at the anterior/posterior compartment boundary of the wing imaginal disc. This Ihog spatial discontinuity may contribute to classically defined cell segregation and lineage restriction at the anterior/posterior wing disc compartment boundary, as suggested by our observations that Ihog activity mediates aggregation of otherwise non-adherent cultured cells and that loss of Ihog activity disrupts wing disc cell segregation, even with downstream genetic rescue of Hedgehog signal response.

## Abstract (web summary)

The range of Hedgehog (Hh) signalling in the *Drosophila* imaginal disc is limited by Hh/receptor degradation at the anterior/posterior (A/P) compartment boundary. Here, the authors show that degradation is mediated by co-receptor proteins Ihog/Boi, whose cell adhesion functions also contribute to A/P cell segregation.

## Introduction

The Hedgehog (Hh) signaling pathway patterns a variety of organs and tissues in metazoan embryos^1–4^. Post-embryonically, the Hh pathway also functions homeostatically in processes such as tissue maintenance and regeneration, acting on tissue stem or progenitor cells^5–9^. Defective Hh signaling during development thus leads to patterning defects such as disrupted segmentation in *Drosophila* ^10^ or holoprosencephaly^11^ and other malformations in humans. Post-embryonic dysregulation of Hh pathway activity can result in proliferative conditions such as malignant tumors^12^ or in tissue degeneration^13^.

The secreted Hh ligand is synthesized as a precursor protein that undergoes cleavage and cholesterol modification via auto-processing, followed by further lipid modification through covalent attachment of palmitate^14^. Release of the mature, dually lipid-modified Hh ligand (HhNp) from cells then requires Dispatched^15, 16^, and also involves other protein and lipoprotein components^17–25^. The Hh protein is typically released from a localized source, and can then induce concentration-dependent cellular differentiation or proliferation responses in cells of surrounding tissues and structures.

In insects and vertebrates, transcriptional upregulation of the receptor component, Patched (Ptc), is a direct response to Hh signaling^26–30^. In *Drosophila* wing imaginal discs, this response occurs exclusively in anterior (A) compartment cells, whereas Hh is produced and secreted exclusively in posterior (P) compartment cells, from where it spreads toward the A compartment^26, 27, 31^. Cells of the A and P compartments do not intermingle but remain segregated within the disc, separated by a smooth boundary that does not correspond to any morphological features^32–34^. This classically defined lineage restriction between cells of the A and P compartment depends largely on the response to the Hh signal exclusively in A cells and is postulated to result from differences in A and P cell affinities^35, 36^. However, the identity of the gene(s) contributing to distinct A and P cell affinities is unknown.

Hh response does not occur in P compartment cells because critical components of the Hh pathway, such as the transcriptional effector Cubitus interruptus (Ci), are not expressed^37^. Cells of the A compartment in contrast express Ci and other pathway components, such as Ptc, which suppresses Smoothened (Smo) activity in the absence of Hh. In A compartment cells located close to the P compartment source of Hh protein, response to the Hh signal stabilizes and activates Smo^38^, and both suppresses formation of Ci repressor and stimulates formation of the activator form of Ci, thus triggering an increase in the transcription of target genes such as *ptc* and decapentaplegic (*dpp*) in a stripe of A cells at the A/P compartment boundary^26, 27, 31, 39, 40^. Moreover, this transcriptional stimulation of Ptc results in a second function of the Hh receptor beyond Smo regulation, namely, the sequestration of Hh and restriction of Hh movement by internalization of Hh through endosomes followed by lysosome degradation^40, 41^.

Although Patched has long been known as an essential component of the *Drosophila* Hedgehog receptor^39, 40^, more recent work shows that the *Drosophila* Hh receptor complex must also include Ihog (Interference Hedgehog) or its close relative Boi (Brother of Ihog) for Hh binding and biological response^42–48^, as well as for sequestration of the Hh protein to limit long-range signaling^42, 43, 49, 50^. The *Drosophila* Ihog and Boi proteins, as well as the related mammalian proteins CAM-related/downregulated by oncogenes (Cdo) and Brother of CDO (Boc)^51^, are type I single-span transmembrane proteins with four or five extracellular immunoglobulin (Ig) domains, two or three extracellular repeats of fibronectin type III (FNIII) domains, and cytoplasmic sequences of unknown structure or function. Our previous biochemical and structural studies showed that the first FNIII domain (Fn1) of Ihog/Boi directly contacts HhN^45, 46^, whereas Fn2, the second FNIII domain of Ihog/Boi, contacts Ptc^43^. The mammalian members of the Ihog family, Cdo and Boc, both contribute to Hh signaling^45, 52–54^ by binding to mammalian Hh proteins via a non-orthologous FNIII repeat^45, 52, 55^.

Although the requirement for Ihog/Boi for response to Hh has been amply confirmed^42–44, 48^, some authors have been unable to observe a role for Ihog/Boi in Hh protein sequestration^56^. Here, we begin by confirming the role of Ihog/Boi in Hh sequestration under physiological conditions. We then explore the mechanism by which Ptc and Ihog/Boi jointly contribute to sequestration of the Hh protein ligand. We identify a post-transcriptional process in which reciprocal regulation of Ihog/Boi and Ptc controls their joint internalization and lysosome degradation upon Hh binding. Remarkably, despite spatially uniform transcription of *ihog* and *boi* genes, this Hh-induced receptor clearance results in reduced levels of Ihog/Boi protein in a stripe of cells at the A/P compartment boundary of the wing imaginal disc. Given that Ihog/Boi proteins resemble typical cell adhesion molecules, we tested for activity in cell–cell adhesion and found that Ihog/Boi indeed mediate aggregation of otherwise non-adhesive cultured cells. In addition, we find that loss of Ihog activity can disrupt A/P cell segregation and lineage restriction, even with downstream genetic rescue of Hedgehog signal response.

## Results

### Ihog/Boi is absolutely required for Hh sequestration

Previously, we reported that Ihog/Boi-expression is required for sequestration of Hh to limit its range of action. In their original work defining the phenomenon of sequestration, Chen and Struhl^40^ established that clones lacking *smo* function on the A side of the A/P boundary show increased expression of endogenous Hh target genes (such as *ptc* or *dpp*) in wild-type cells just anterior to the *smo* mutant clone, due to loss of Hh-induced *ptc* expression within the *smo* mutant clone. Chen and Struhl^40^ also noted that upregulated expression of *ptc* through downstream pathway activation by additional mutation of the cAMP-dependent protein kinase 1 (PKA-C1) within *smo* mutant clones restores sequestration of Hh, as indicated by lack of increased expression of endogenous Ptc in wild-type cells immediately anterior to the *smo*; *pka-C1* clones. We confirm this finding (Supplementary Fig. 1A), but also note that Ptc expression persists on the anterior side of *pka-C1* clones that also lack Ihog/Boi, at an abnormally large distance from the Hh-expressing posterior cells (Supplementary Fig. 1B, E, F). Taken together, these results confirm our previous conclusion that Ihog/Boi-expression is required for Hh sequestration to limit its range of activity, and that expression of the Ptc protein at physiological levels alone is not sufficient for this sequestration activity^43^ (see Supplementary Discussion for more in depth discussion and for analysis of the work of Camp et al., who failed to observe a requirement of Ihog/Boi for sequestration). In the work presented below, we explore the mechanism underlying reciprocal regulation of Ptc and Ihog/Boi in Hh sequestration.

### Ihog promotes Ptc-mediated Hh endocytosis in vitro

We previously demonstrated that Ihog/Boi proteins interchangeably function to interact directly with Ptc, and that Ihog/Boi activity is required for presentation of Ptc on the cell surface. We also found that Ihog/Boi activity and Ptc are both required for high-affinity Hh binding^43^. This crucial role of Ihog/Boi in binding of Ptc to Hh adequately explains why Ihog/Boi activity is indispensable in transducing the Hh signal. However, the process of Ptc-mediated Hh sequestration also requires elimination of Ptc-bound Hh from the cell surface by endocytosis^40, 41^. We therefore reasoned that if Ihog/Boi activity is required for Hh sequestration, it may also be essential for Ptc-mediated Hh endocytosis.

**Fig. 1:**
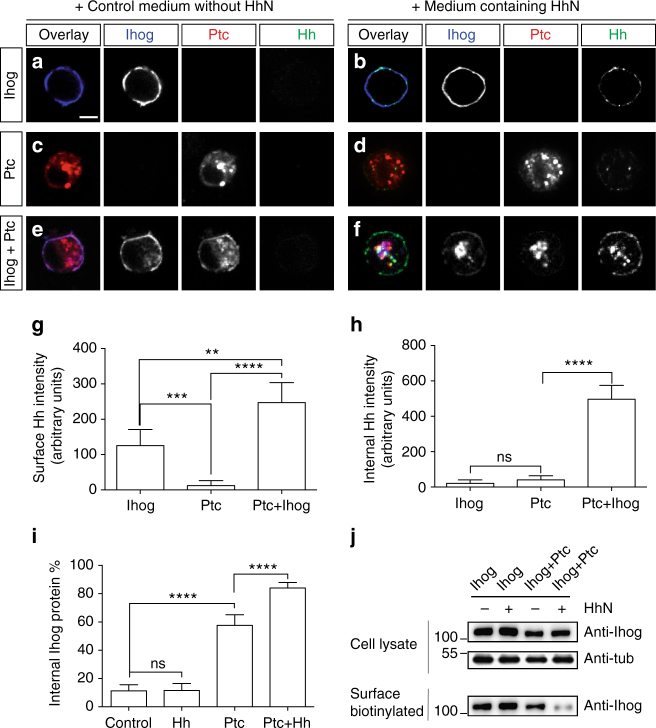
Subcellular localization of Ihog, Ptc and Hh in S2R+ cells. **a**–**f** Confocal microscope images showing localization of HhN (green), Ihog (blue), and Ptc (red) proteins. *Drosophila* S2R+ cells were transfected for expression of Ptc and/or Ihog, followed by treatment of control medium without Hh (**a**, **c**, **e**) or medium containing HhN (**b**, **d**, **f**) for 1 h at room temperature. Scale bar, 5 µm. **g**, **h** Quantification of surface HhN intensity (**g**) and internalized HhN intensity (**h**) from cells transfected with Ptc and/or Ihog. Each bar shows the mean ± s.d. of fluorescence from *n* = 20 cells, and representative images are shown in **b**, **d**, **f**. **i** Quantification of Ihog protein intensity both on the cell surface and inside of the cells. Internal Ihog protein% = Ihog internal intensity/(Ihog internal intensity + Ihog surface intensity)%. Each bar shows the mean ± s.d. from *n* = 20 cells, and representative images are shown in **a**, **b**, **e**, **f**. Two-tailed unpaired Mann–Whitney *U*-test was used for statistical analysis. ns not significant. **P < 0.01. ***P < 0.001. ****P < 0.0001. **j** S2R+ cells transfected for expression of Ptc and/or Ihog were labeled by surface biotinylation. Immunoblots in the top panels show 5% of the biotinylated cell lysate, and the bottom panel shows the biotin-labeled proteins recovered by Streptavidin agarose beads. Co-expression with Ptc and treatment of HhN reduced levels of Ihog on the cell surface

To test this hypothesis, we first asked whether Ihog can promote Ptc-mediated Hh endocytosis in vitro. To this end, we transfected cultured *Drosophila* S2R+ cells with Ihog or Ptc alone, and in combination. After incubating the transfected cells with HhN-containing medium for 1 h at room temperature to allow for internalization, we immunostained the cells with a protocol allowing detection of both surface and internalized protein (“Methods”). Consistent with previous observations^43^, we found that the amount of surface HhN markedly increased upon co-transfection of Ptc and Ihog as compared to Ptc alone (compare Fig. 1f to d; quantification in Fig. 1g). Importantly, the presence of Ihog also significantly augmented internalization of HhN in Ptc-expressing cells (compare Fig. 1f to d; quantification in Fig. 1h). In this experiment, we also noticed that the Ihog protein alone mainly localized to the cell surface and, although bound to HhN, was not able to internalize HhN in the absence of Ptc (Fig. 1b; quantification in Fig. 1g and h). Consistent with the overlapping role of Ihog and its close relative Boi as Hh co-receptors, we found co-expression of Boi with Ptc also increased HhN internalization in S2R+ cells. Altogether, our data suggest Ihog proteins are able to promote Ptc-mediated Hh internalization in vitro.

### Ihog is essential for Ptc-mediated Hh endocytosis in vivo

**Fig. 2:**
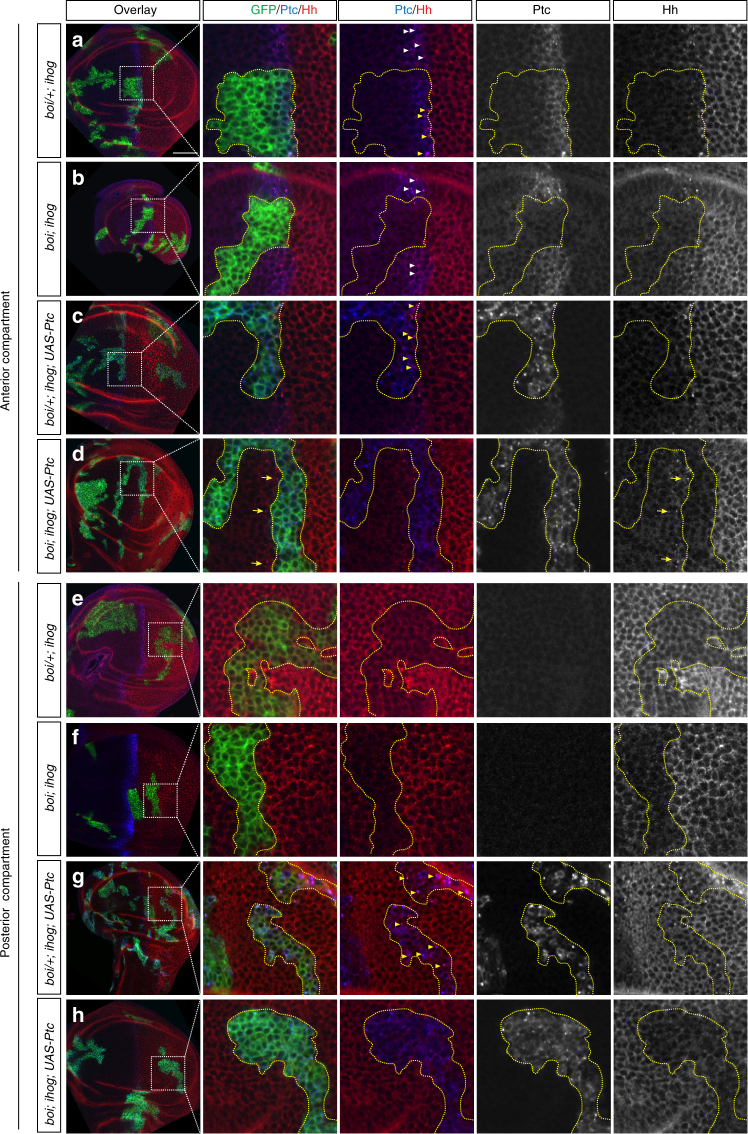
Ihog/Boi is required for Ptc-mediated Hh endocytosis in vivo. **a**–**h** Wing discs were immunostained for GFP (green), Ptc (blue), and Hh (red). MARCM clones (indicated by expression of mCD8GFP) lacking *ihog* were induced in *boi* heterozygous (*boi*/+; *ihog* in **a**, **c**, **e**, and **g**) or *boi* hemizygous (*boi*; *ihog* in **b**, **d**, **f**, and **h**) larvae. **c**, **d**, **g**, **h** MARCM clones simultaneously express a *UAS-Ptc* transgene. **a**–**d** In Hh-receiving cells of the A compartment adjacent to the boundary, Hh protein internalized with upregulated Ptc protein in wild-type cells (**a** white arrow heads) or cells within clones with a single wild-type dose of *boi* (**a** yellow arrow heads), but not in *boi*; *ihog* double mutant clones (**b** yellow outline). Ectopic expression of Ptc in *boi*; *ihog* clones showed no rescue of Hh internalization (**d** yellow outline). Note that HhN traveled through the *UAS-Ptc*-expressing *boi*; *ihog* double mutant clones and accumulated in wild-type cells immediately anterior to the mutant clone (**d**, yellow arrows). **e**–**h** In Hh-secreting cells of the P compartment, Hh protein internalized with ectopically expressed Ptc protein within clones with a single wild-type dose of *boi* (**g**), but not in *boi*; *ihog* double mutant clones (**h**). Control *ihog* clones in *boi* heterozygous or *boi* hemizygous animals not expressing *UAS-Ptc* transgene showed no internalization of Hh (**e**, **f**). Scale bar, 50 µm

Encouraged by the ability of Ihog/Boi to promote Ptc-mediated Hh internalization in S2R+ cells, we examined whether Ihog/Boi is required for Ptc-mediated Hh endocytosis in the wing imaginal disc. Hh expression in the disc is limited to cells of the posterior (P) compartment, which do not respond to the Hh signal. Target gene expression is thus restricted to a stripe of adjacent cells within the anterior (A) compartment. Given that the *ptc* gene not only encodes a component of the Hh receptor but is also a transcriptional target for Hh signaling, its expression serves as a reporter of Hh response and its highest level of expression is in a stripe of A cells immediately adjacent to the A/P compartment boundary (Fig. 2a). The Ptc protein in this stripe of A cells binds and internalizes Hh ligand secreted from the P cells. Wild-type wing disc cells (with one or two wild-type alleles of *ihog/boi*) thus exhibit punctate Hh staining co-localized with Ptc in intracellular vesicles (arrow heads, Fig. 2a). A similar Hh protein distribution has been described previously^26, 57^. In contrast, we found that removing all function of both *ihog* and *boi* eliminated punctate Hh staining in the A cells at the A/P boundary (Fig. 2b). As Ptc protein is not expressed in clones lacking both Ihog and Boi, the loss of internalized Hh we noted could be due to loss of Ptc^43^ (Fig. 2b). To determine whether Ihog/Boi proteins are required for Hh endocytosis in the presence of Ptc, we activated ectopic Ptc expression by MARCM in wing disc cells and compared cells with and without Ihog/Boi function (Fig. 2c, d). We observed HhN puncta in *boi/+*; *ihog* clones expressing Ptc (Fig. 2c; blue arrows), but not in Ptc-expressing cells lacking both Ihog and Boi (Fig. 2d). We note that the presence of a single wild-type *ihog/boi* allele sufficed to prevent a defect of Hh internalization, in either the presence or absence of ectopic Ptc expression (Fig. 2a, c), consistent with previous findings that a single wild-type allele of *ihog/boi* suffices to provide functional activity^43^. These results confirm that Ihog/Boi-expression is required for Ptc-mediated endocytosis of Hh in A cells adjacent to the A/P boundary, and that expression of the Ptc protein alone is not sufficient for internalization.

Consistent with the previous observations^42, 58^, we also found that Ihog and Boi are required to retain Hh on the surface of P compartment cells in the wing imaginal disc (Hh is present on the surface of cells within clones in Fig. 2e (*boi/+*; *ihog*); compare to clones in Fig. 2f (*boi*; *ihog*)). This activity is dosage-dependent, as reducing Ihog/Boi activity by removing a single wild-type *boi* allele caused a partial loss of Hh on the surface of P cells (compare cells within and outside of clones in Fig. 2e, f). As P cells do not express *ptc* ^59, 60^, Hh retention on the surface of P cells is apparently Ptc-independent, and these observations are consistent with our in vitro data showing that HhN was retained by cells transfected to express only Ihog and not Ptc, and that surface-bound HhN was not internalized (Figs. 1a, 2e, and 2f). However, upon ectopic expression of Ptc in P compartment cells, Ptc and Hh were found in punctate structures in cells that contain both proteins^61^. Thus, we wondered whether Ihog and Boi are also required for the ectopic Ptc-mediated Hh internalization in P cells. To answer this question, we ectopically expressed Ptc in P cells by MARCM and found that Ptc induced Hh internalization in P cells with a single dose of wild-type *boi* indicated by the punctate co-localization of Ptc and Hh ((*boi/+*; *ihog* clones; Fig. 2g), but not in cells when both endogenous *ihog* and *boi* are removed (Fig. 2h). Our evidence thus shows that Ihog/Boi is required for Ptc-mediated Hh endocytosis in Hh-responding A cells, but also for endocytosis in Hh-producing P cells when Ptc is ectopically expressed.

### Ihog mediates Hh-induced Ptc degradation

Ptc limits the Hh gradient by internalizing Hh, and targeting it for lysosomal degradation^41^. Conversely, Hh stimulation has been reported to cause degradation of Ptc^38, 41, 62, 63^. As Ihog/Boi activity is required both for Ptc binding^43^ and internalization of Hh (see above; Figs. 1 and 2), we examined the role of Ihog/Boi in Hh-dependent change of Ptc subcellular localization and degradation.

**Fig. 3:**
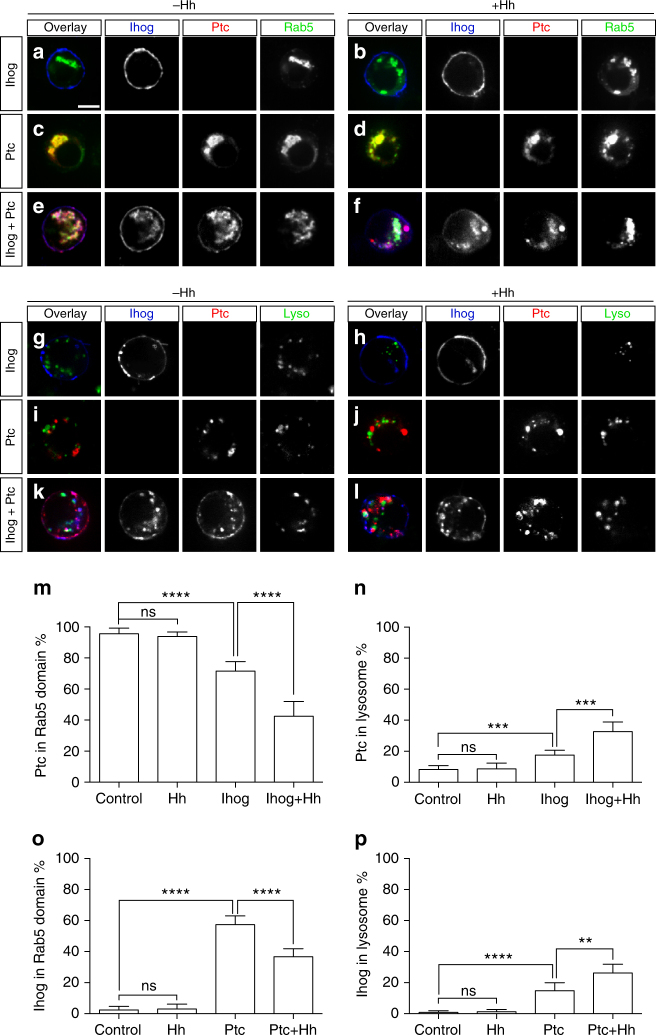
Reciprocal regulation of Ihog and Ptc in Hh-induced receptor degradation. **a**–**f** Confocal microscope images showing localization of Rab5 (green), Ihog (blue), and Ptc (red) proteins in fixed and immunostained *Drosophila* S2R+ cells, which were transfected for expression of Actin-Gal4, UAS-Rab5-GFP, Ptc and/or Ihog, and treated with control medium or medium containing HhN for 1 h at room temperature. **g**–**l** Confocal microscope images showing localization of Ihog (blue), Ptc (red), and LysoTracker Deep Red (green) in live *Drosophila* S2R+ cells, which were transfected for expression of Ihog-GFP, Ptc-mCherry alone or in combination, and treated with control medium or medium containing HhN for 1 h at room temperature. Scale bar, 5 µm. **m**–**p** Quantification of Ptc intensity (**m**, **n**) or Ihog intensity (**o**, **p**) in early endosomes marked by Rab5 (**a**–**f**) or lysosomes stained with LysoTracker (**g**–**l**). Each bar shows the mean ± s.d. of fluorescence from *n* = 20 cells, and representative images are shown in **a**–**l**. Two-tailed unpaired Mann–Whitney *U*-test was used for statistical analysis. ns not significant. **P < 0.01. ***P < 0.001. ****P < 0.0001

To examine Ihog effects on subcellular localization of Ptc, we co-transfected S2R+ cultured cells for expression of Ptc and Ihog, alone or in combination, followed by incubation with medium containing HhN. When transfected alone, Ptc localized primarily in intracellular vesicles^43^, likely early endosomes as indicated by co-localization with Rab5 staining and absence of co-localization with lysosomal staining (Fig. 3c, i; quantification in Fig. 3m, n). In the absence of Ihog co-expression, the early endosome localization of Ptc was not markedly changed upon Hh treatment (Fig. 3d, j; quantification in Fig. 3m, n). In contrast, Hh treatment of cells co-expressing Ihog markedly shifted localization of a significant fraction of Ptc from the Rab5-positive early endosome into lysosomes (Fig. 3f, l; quantification in Fig. 3m, n). Expression of Ihog protein thus contributes to lysosome targeting of Hh and Ptc.

We next compared endogenous Ptc protein levels in S2R+ cells in the presence or absence of HhN, as a function of RNAi directed against *ihog/boi* mRNA. We observed that Ptc protein in cells transfected with control dsRNA was undetectable after 4 h of HhN treatment (Supplementary Fig. 2A, compare lanes 2 and 1), whereas HhN-triggered degradation of Ptc was much less efficient in cells transfected with dsRNA against *ihog* and *boi* (Supplementary Fig. 2A, compare lanes 4 and 2). Furthermore, accumulation of phosphorylated Smo was induced rapidly by HhN stimulation (Supplementary Fig. 2A, compare lanes 2 and 1) or by Ptc RNAi regardless of Hh stimulation (Supplementary Fig. 2A, lanes 5 and 6), whereas combined RNAi against *ihog* and *boi* in addition to stabilizing Ptc also reduced Smo phosphorylation in the presence of HhN (Supplementary Fig. 2A, compare lanes 4 to 2, 5 and 6). The mechanism of Hh-induced and Ihog-dependent Ptc degradation would appear to involve ubiquitinylation, as the level of Ptc modification by ubiquitin was markedly increased by Ihog-expression and HhN-stimulation (Supplementary Fig. 2B).

**Fig. 4:**
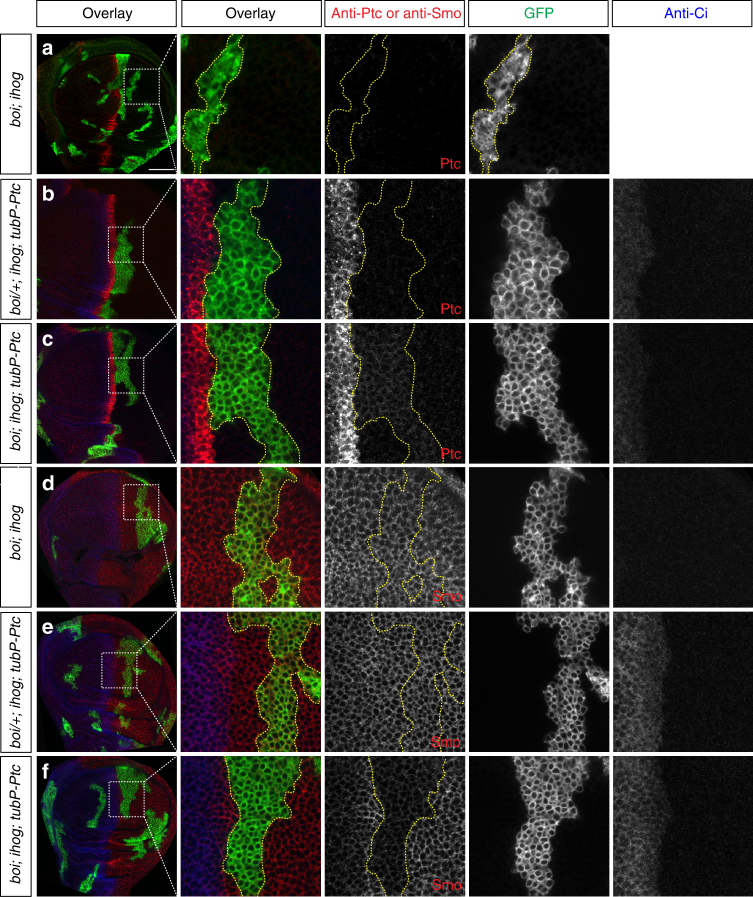
Ihog/Boi mediates Hh-dependent degradation of Ptc in vivo. **a**–**f** Wing discs were immunostained for GFP (green), Ci (blue), and Ptc (**a**–**c**) or Smo (**d**–**f**) as indicated (red). MARCM clones (indicated by expression of mCD8GFP) lacking *ihog* were induced in *boi* heterozygous (*boi/+*; *ihog* in **b**, **e**) or *boi* hemizygous (*boi*; *ihog* in **a**, **c**, **d**, and **f**) larvae. **b**, **c**, **e**, **f** MARCM clones were generated in animals constitutively expressing a low level of Ptc protein in all cells from the transgene *tubP-Ptc* (*Tubα1P-Ptc-Tubα1 3′UTR*)^40^. In Hh-secreting cells of the P compartment, Ptc protein only accumulated within *boi*; *ihog* double mutant clones (**c**), and not in clones with a single wild-type dose of *boi* (**b**). Correspondingly, endogenous Smo protein levels were markedly reduced in *boi*; *ihog* clones with stabilized Ptc proteins (compare **f** and **e**). Control *boi*; *ihog* clones in animals not carrying the *tubP-Ptc* transgene showed no accumulation of Ptc (**a**) and no loss of Smo (**d**). Scale bar, 50 µm

The incomplete effects of RNAi treatments on Ptc degradation and phosphorylation of Smo are likely due to the fact that RNAi treatments in cultured cells (Supplementary Fig. 2A) are seldom fully efficient. To circumvent this difficulty and to examine degradation of Ptc in vivo, we created mutant clones lacking both *ihog* and *boi* in imaginal discs that constitutively express a low level of Ptc protein from the transgene, *tubP-Ptc*, in all cells^40^. Because the endogenous *ptc* gene is not expressed in posterior compartment cells and because Hh expression in P cells is high, no Ptc protein from the *tubP-Ptc* transgene could be detected in wild-type cells; Ptc protein similarly was undetectable in clones with a single dose of wild-type *boi* (*boi/+*; *ihog* clones; Fig. 4b). In cells lacking both Ihog and Boi (*boi*; *ihog* clones), however, low levels of Ptc protein produced by the *tubP-Ptc* transgene were detectable (Fig. 4c), indicating that Ihog/Boi function is required in vivo for Hh-induced Ptc degradation. As would be expected, Smo protein levels are markedly reduced in *boi*; *ihog* clones with stabilized Ptc proteins (compare Fig. 4f to e). No Ptc was detectable in control posterior *boi*; *ihog* clones lacking the *tubP-Ptc* transgene (Fig. 4a), and, correspondingly, no loss of Smo was detected (Fig. 4d).

### Endocytosis and degradation of Ihog together with Hh/Ptc

Given the critical role of Ihog/Boi activity in Ptc-mediated Hh internalization and the occurrence of Hh-induced degradation of Ptc, we considered the possibility that Ihog itself may be internalized and degraded in a Ptc-dependent and Hh-dependent manner. Indeed, we noted by immunostaining that Ihog is internalized in S2R+ cultured cells co-expressing Ptc and treated with HhN (compare Fig. 1e, f; quantified in Fig. 1i), and furthermore that surface biotinylation of Ihog was reduced in cells co-expressing Ptc and treated with HhN (Fig. 1j). The internalized Ihog proteins were found both in Rab5-positive early endosomes (Fig. 3f; quantified in Fig. 3o) and lysosomes (Fig. 3l; quantified in Fig. 3p). Thus, Ihog protein itself was internalized with Ptc in a HhN-dependent manner. In the absence of externally added HhN, we also observed a greater internalization of Ihog in S2R+ cells co-expressing Ptc (compare Fig. 1e with a, Fig. 3e with a, and Fig. 3k with g). In addition, we noted that the levels of Ihog in the cell lysate also decreased upon co-expression with Ptc (Fig. 1j). Given that no endogenous Hh is expressed in these cells^64^, Ptc degradation associated with its normal turnover^65^ may be responsible for causing both Ihog internalization and degradation.

**Fig. 5:**
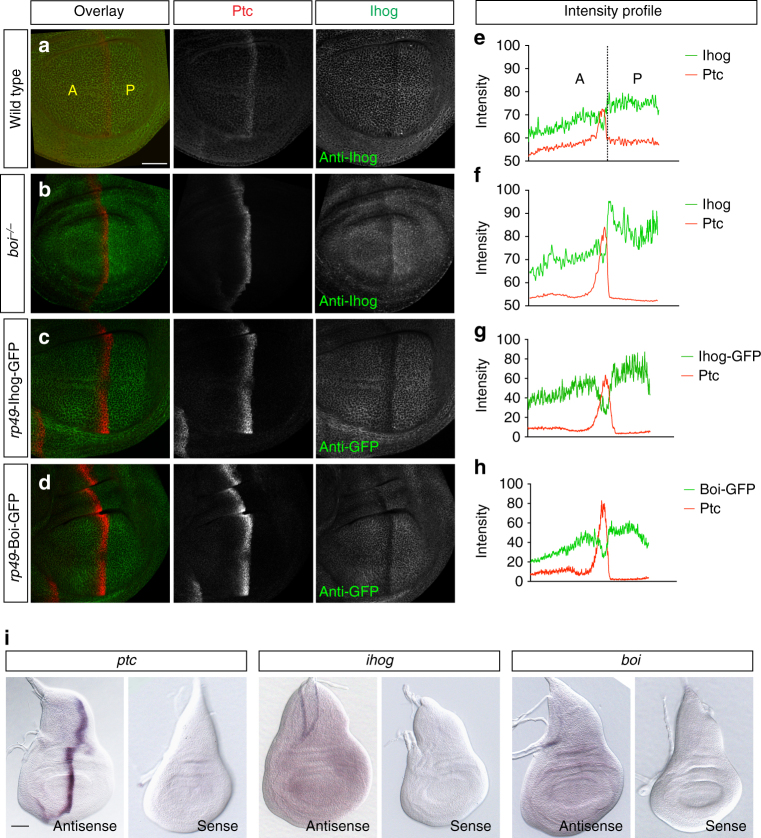
Ihog is post-transcriptionally downregulated at AP boundary. **a**, **b** Wing imaginal disc from wild-type (**a**) or in *boi* homozygous (**b**) larvae were immunostained for Ihog (green) and Ptc (red). Lower Ihog levels were detected in the A compartment, and lowest levels occur in the Ptc-expressing cells adjacent to the A/P compartment boundary. **c**, **d** Wing imaginal discs from larvae carrying *rp49-Ihog-GFP* or *rp49-Boi-GFP* immunostained for GFP (green) and Ptc (red). Scale bar, 50 µm. **e**–**h** Plotted pixel intensities of Ptc and Ihog or GFP as a function of A/P position. **i** Whole mount in situ on third instar wing imaginal discs to show the distribution of *ptc*, *ihog*, and *boi* transcripts. Scale bar, 50 µm

Consistent with a role for Ptc in mediating Hh-dependent degradation of Ihog, antibody staining of the wing imaginal disc revealed that Ihog protein levels are inversely correlated with Ptc protein levels, namely, higher in the P compartment, lower in the A compartment, and at their lowest in a region coinciding with the stripe of high-level Ptc protein expression in A cells at the A/P compartment boundary^42^ (Fig. 5a, e; Supplementary Fig. 3). This Ihog protein expression pattern is not due to re-localization of Ihog proteins in the columnar epithelium of the *Drosophila* wing disc, as the same expression pattern of Ihog protein is observed in each single focus plane from the apical to the basal surface through the entire columnar epithelium (Supplementary Fig. 4; Supplementary Movies 1–3). Moreover, this Ihog protein expression pattern is not due to Hh-induced downregulation of the *ihog* transcripts, as in situ hybridization revealed a uniform expression pattern of the *ihog* transcripts in the wing imaginal disc (Fig. 5i). To confirm that the reduced level of Ihog protein at the A/P compartment boundary is indeed due to a post-transcriptional phenomenon, we also generated a transgene encoding wild-type Ihog tagged with green fluorescent protein (GFP) under the control of the ubiquitous promoter *rp49* (*L*>*Ihog-GFP*)^66^ and quantified its abundance in various regions of the wing imaginal disc (Fig. 5c, g). Low levels of Ihog-GFP (<1.5-fold endogenous Ihog) are expressed ubiquitously throughout the organism from this transgene. However, in wing discs from flies with *L*>*Ihog-GFP*, the abundance of Ihog-GFP was decreased in A cells at the A/P compartment boundary, precisely in those cells where transcription of the endogenous *ptc* gene is induced (Fig. 5c, g). Similar to *ihog*, we also found that the *boi* transcripts in the wing imaginal disc were uniform (Fig. 5i) and that the expression levels of Boi-GFP protein were reduced in A cells at the A/P boundary upon introduction of a *L*>*Boi-GFP* transgene (Fig. 5d, h). Remarkably, this negative correlation between Ihog and Ptc protein levels becomes more evident in wing discs from *boi* mutant larvae (Fig. 5b, f; Supplementary Figs. 3–5; Supplementary Movies 1–6). This is likely due to the overlapping roles of Ihog and Boi^43^, and consequent enhancement of the Hh/Ptc-induced Ihog degradation caused by loss of Boi.

**Fig. 6:**
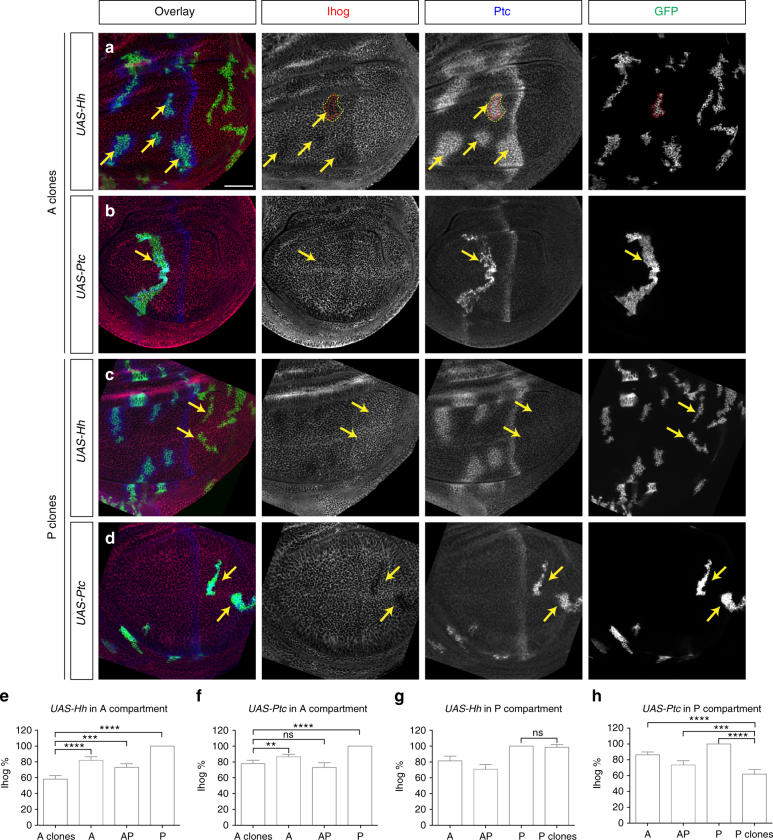
Hh/Ptc-dependent modulation of Ihog protein levels in wing imaginal discs. Wing imaginal discs were immunostained with antibodies against Ihog (red), Ptc (blue), and GFP (green) proteins. **a**, **c** Activation of UAS-driven Hh expression in clones marked by expression of GFP (green). Ptc protein levels are increased in Hh-expressing cells and in nearby cells within the A compartment (arrows in **a**), but not in the P compartment (arrows in **c**). Reduced Ihog (red) levels are consistently detected in cells with elevated Hh and Ptc expression (arrows in **a**). Note that the reduction of Ihog levels in A compartment extends beyond cells in the clone that express Hh (red lines in **a**), but coincides with cells showing Hh-induced expression of high levels of Ptc (yellow lines in **a**). **b**, **d** Activation of UAS-driven Ptc expression (blue) in clones marked by expression of GFP (green). Ihog is also slightly reduced in clones with elevated Ptc expression when located in the A compartment (arrow in **b**) far away from Hh-producing cells. Ihog protein levels are markedly reduced in the Ptc-expressing clones located in the P compartment where Hh is highly expressed (arrows in **d**). Scale bar, 50 µm. **e**–**h** Quantification of Ihog intensity in clones marked by GFP and different compartments determined by Ptc staining in the wing discs. Ihog intensity at different location is normalized by that of P compartment of the same wing disc. Each bar shows the mean ± s.d. of normalized Ihog intensity from *n* = 10 wing discs, and representative images are shown in **a**–**d**. Two-tailed unpaired Mann–Whitney *U*-test was used for statistical analysis. ns, not significant. **P < 0.01. ***P < 0.001. ****P < 0.0001

To further investigate whether Ptc and Hh expression can indeed influence Ihog protein levels in vivo, we generated GFP-marked clones of cells expressing Hh from a Gal4-responsive promoter; as A cells are Hh-responsive, A clones responded to Hh stimulation by expressing high levels of Ptc, and these cells indeed displayed reduced Ihog protein levels (Fig. 6a, e). No change in Ihog protein levels was noted in similar P clones (Fig. 6c, g), where cells normally express Hh but lack Ci and consequently are not responsive to the Hh signal and therefore do not express Ptc. We next generated GFP-marked clones of cells expressing Ptc. We found that Ptc expression indeed reduced Ihog protein levels in the P compartment, where Hh but not Ptc is normally expressed (Fig. 6d, h). We noted that the reduction of Ihog levels in posterior cells clonally activated to express Ptc is restricted to cells within the clone (Fig. 6d, h), whereas Ihog reduction in the anterior extends beyond cells clonally activated for expression of Hh (Fig. 6a). We interpret this to be due to Hh stimulation and Ptc expression in cells beyond the boundary of the Hh-expressing clone. These data indicate that internalization and degradation of Ihog protein is triggered cell-autonomously by expression of Ptc, which can however be stimulated in a cell non-autonomous manner by the secreted Hh protein signal.

We also generated Ptc-expressing clones in the anterior, far from Hh-expressing posterior cells, by expression of the *UAS-Ptc* transgene (Fig. 6b, f). Consistent with our cultured cell studies (Fig. 1), we noticed Ihog levels were slightly reduced within these Ptc-expressing clones, far away from a source of Hh ligand, suggesting that some downregulation of Ihog proteins can be mediated by Ptc alone, in the absence of Hh ligand.

### Ihog mediates aggregation of *Drosophila* S2 cells

**Fig. 7:**
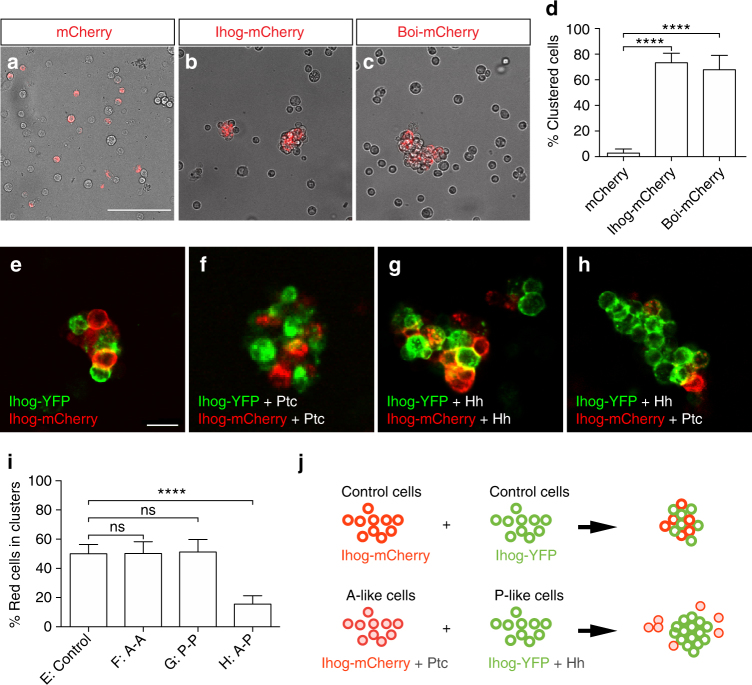
S2 cells expressing Ihog and Ptc segregate from those expressing Ihog and Hh. **a**–**c** *Drosophila* non-adhesive S2 cells were transfected for expression of mCherry (**a**), Ihog-mCherry (**b**), or Boi-mCherry (**c**). Note the bright field channel image shows both transfected and un-transfected S2 cells. S2 cells aggregate upon expression of Ihog-mCherry and Boi-mCherry, indicated by the cluster formation of transfected cells (mCherry expressing) under the exclusion of un-transfected cells. In contrast, the cells transfected by mCherry alone do not aggregate. Scale bar, 100 µm. **d** The aggregation effect was quantified as the ratio of transfected cells within a cluster to total transfected cells. Each bar shows the mean ± s.d. from *n* = 20 different images, and representative images are shown in **a**, **b**, **c**, **e**–**h**. S2 cells were transfected separately with plasmids expressing Ihog-YFP/mCherry, Ihog-YFP/mCherry and Hh, or Ihog-YFP/mCherry and Ptc. Cells were dissociated by trypsin treatment and then mixed 4 h to allow aggregation to occur. Scale bar, 20 µm. **i** Ratio of red (mCherry expressing) cells from within a cluster to total transfected cells in **e**–**h**. Each bar shows the mean ± s.d. from *n* = 20 different images, and representative images are shown in **a**, **b**, **c**, **j**. Model of differential aggregation of S2 cells expressing Ihog/Hh and Ihog/Ptc. Two-tailed unpaired Mann–Whitney *U*-test was used for statistical analysis. ns not significant. ****P < 0.0001

The reduced level of Ihog/Boi proteins at the A/P compartment boundary is intriguing in light of the long-known lineage restriction between A and P compartment cells^32–34^. Ihog/Boi proteins resemble cell adhesion molecules: both are type I single-span transmembrane proteins with 4 immunoglobulin and 2 fibronectin type III extracellular domains. However, whether Ihog/Boi proteins directly function in cell adhesion is not known. To address this possibility, we assessed the consequences of overexpressing Ihog or Boi in *Drosophila* S2 cells, which are inherently non-adhesive and have been used to demonstrate the adhesive properties of other membrane proteins^67^. Notably, we found that S2 cell cultures co-transfected with mCherry and either Ihog-expression or Boi-expression constructs formed multi-cellular aggregates (Fig. 7b–d), whereas S2 cell cultures transfected with mCherry alone did not (Fig. 7a, d). Within each experiment, only the cells transfected to express Ihog/Boi participated in aggregate formation and un-transfected cells were excluded (Fig. 7b–d), suggesting that aggregation is homophilic and requires expression of Ihog or Boi on each interacting cell. Ihog/Boi proteins, in addition to functioning as Hh receptor components^43^, thus also appear to function in cell adhesion.

### Modulation of Ihog protein levels causes S2 cell segregation

The expression levels of Ihog and Boi are lowest in anterior cells at the A/P compartment boundary as a result of Hh-triggered receptor internalization and degradation (Fig. 5). As a small difference in the abundance or activity of a single species of homophilic cell adhesion molecule between two interspersed populations of cells is predicted to cause segregation of the two populations^68^, it is conceivable that the modulation of the level of Ihog proteins at the A/P boundary may contribute to segregation of and lineage restriction between A and P cells. To simulate this situation, two populations of S2 cells were transfected separately (Fig. 7e–h): one with constructs for expression of an Ihog-mCherry fusion and Ptc, to mimic A cells adjacent to the A/P compartment boundary (expressing high levels of pathway target genes, including Ptc), and the other with plasmids for expression of an Ihog-YFP fusion and Hh, to mimic Hh-secreting P cells. We note that as S2 cells lack endogenous expression of Ci, this experiment is not affected by transcriptional response but only by the role of Hh receptor binding in cell adhesion.

Forty-eight hours after transfection, cells were dissociated and mixed for 4 h to allow A-like and P-like cells to interact. Our expectation was that degradation of the Ihog/Ptc complex in A-like cells would be induced by Hh produced in P-like cells. We indeed found that P-like cells co-expressing Ihog-YFP and Hh (green) frequently aggregate only with themselves and sort out from the A-like cells co-expressing Ihog-mCherry and Ptc (red) (Fig. 7h, i). In parallel, we measured the expression level of both total (Supplementary Fig. 6A) and surface Ihog proteins (Supplementary Fig. 6B–F) in the S2 cells used in cell-mixing assay before and 4 h after cell mixing. Consistent with what we presented above, the total Ihog protein is selectively reduced after mixing A-like and P-like cells. We indeed found that the Ihog protein localized on the surface of A-like cells was markedly reduced after mixing with P-like cells (Supplementary Fig. 6B, F). The significant difference in level of surface Ihog protein on A-like and P-like cells likely is the driving force for their segregation after mixing. Likewise, a greater decrease of the Ihog protein localized to the cell surface at the AP boundary was observed in vivo when we performed immunostaining using antibodies against the extracellular domain of Ihog in the absence of detergent (Supplementary Fig. 7). This result suggests that a difference in Ihog protein levels at the A/P boundary, especially at the cell surface, may suffice to cause differences in cell affinity in vivo, and could at least partially contribute to A/P cell segregation in wing discs (Fig. 7j).

### Ihog proteins affect A/P cell segregation in vivo

**Fig. 8:**
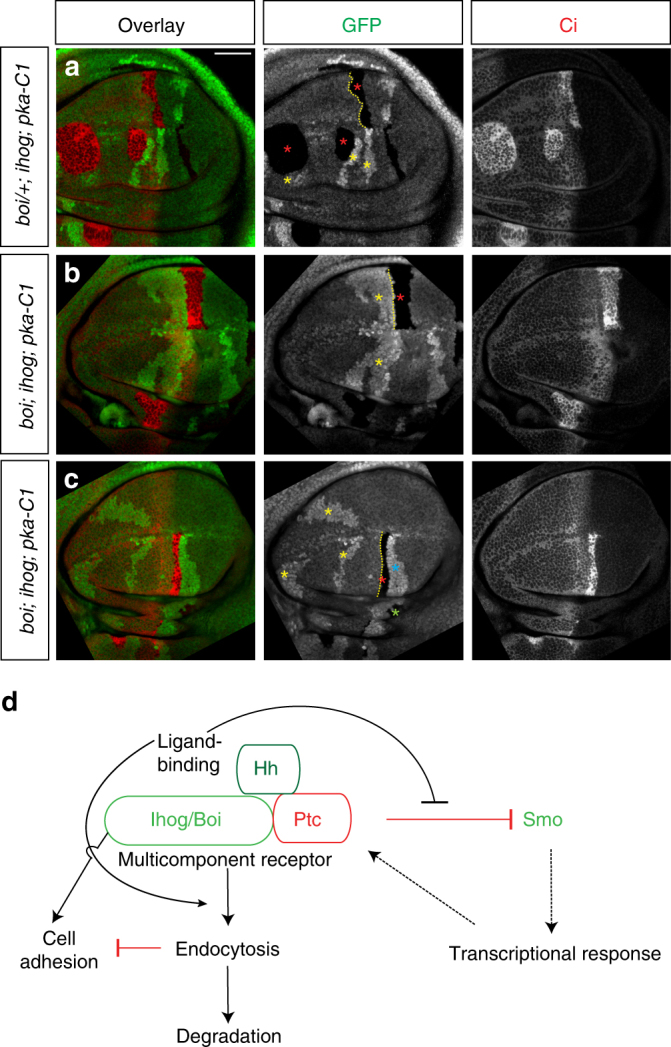
Ihog contributes to AP cell segregation independent of their Hh receptor function. **a**–**c** Each set of panels shows a wing imaginal disc immunostained for GFP (green) and Ci (red). The mutant clones are marked by the absence of GFP expression (red asterisks), and the wild-type sister clones composed of cells that have two copies of GFP and thus are marked by elevated level of GFP expression (yellow asterisks). **a** *pka-C1* mutant A cells (GFP negative, Ci positive, red asterisks) arising from A cells close the A/P boundary mix well with adjacent A cells (dashed yellow line showing a wiggly border). **b** *pka-C1* loss failed to rescue the cell segregation defects of *boi*; *ihog* mutant A cells (GFP negative, Ci positive, red asterisks). Note that *boi*; *ihog* mutant cells form a sharp boundary (yellow line) with adjacent A cells (yellow asterisks). **c** Note that *boi*; *ihog pka-C1* triple mutant clones are absent in the A compartment away from the A/P boundary of the wing pouch even with the presence of three wild-type sister clones (yellow asterisks); these triple mutant clones typically accumulate in a large clone at the A/P boundary (see text). The *boi*; *ihog pka-C1* triple mutant clone originating in the posterior compartment of the wing pouch is indicated with a green asterisk and its wild-type sister clone is indicated by a blue asterisk. Scale bar, 50 µm. **d** Schematic summary of Ihog protein activity. In the absence of Hh, Ihog/Boi interacts with Ptc to form a receptor that is presented on the cell surface^43^. When Hh ligand is present, it binds this receptor and blocks the inhibition of Smo by Ptc, thus permitting activation of downstream pathway components. Binding of the Hh ligand induces ligand:receptor internalization and degradation of the ligand:receptor complex, preventing further action of Hh on distant cells. Ligand-induced receptor clearance also creates a discontinuity in the Ihog protein levels at the A/P compartment boundary, which may contribute to cell segregation and lineage restriction at the compartment boundary by reducing cell adhesion

Loss of Hh responsiveness in large clones of anterior cells results in invasion of posterior territory^35, 36, 40^, and similar behavior for large clones of cells lacking *ihog/boi* function has been previously reported^43, 48^. Large clones of cells lacking *ihog/boi* and located near the A/P border thus were seen to reside within territory normally occupied by posterior compartment cells, even though expression of Ci clearly marked them as originating in the anterior compartment (Supplementary Fig. 8). To investigate whether this abnormal cell segregation behavior is due simply to loss of Hh signal transduction, we generated large clones of cells lacking not only Ihog/Boi but also PKA-C1; the latter causes Hh pathway activation through stabilizing effects on Ci, thus resulting in pathway activation in *ihog/boi* mutant clones^69–73^. We found that, although *pka-C1* mutant A cells with functional Boi protein close to the AP boundary mix well with adjacent A cells (see rough boundary, Fig. 8a), the segregation defects of *boi*; *ihog* mutant A cells were not rescued by *pka-C1* loss, as these triple mutant cell clones (*boi*; *ihog*; *pka-C1*) now form a smooth boundary with adjacent A cells (Fig. 8b, c).

One other unusual feature of these large triple mutant cell clones (*boi*; *ihog*; *pka-C1*) is their relative absence at more anterior locations in the A compartment. Sister clones (twin spots) derived from a single recombinant event are usually located adjacent or close to each other (85% in leg discs, higher in wing discs)^74^, regardless of their location within the disc. We noted this type of pairing where sister clones both retain *boi* ^+^ function (Fig. 8a, sister clones denoted by red and yellow asterisks). In contrast, *ihog* ^+^ clones arising in the A compartment away from the A/P boundary (marked by double GFP expression in the A compartment of the wing pouch—see yellow asterisks in Fig. 8c) were often found unaccompanied by sister clones when these sisters were expected to lack both *ihog* and *boi* function (*boi*; *ihog*; *pka-C1*; marked by absent GFP expression, red asterisks in Fig. 8c; see Supplementary Fig. 10A). Quantification shows a high statistical significance (*p* < 0.001) to the reduced frequency of pairing of such clones (Supplementary Fig. 10B).

Triple mutant clones appear not to have general survival or growth defects, as wing discs containing unpaired clones of double-intensity GFP expression invariably also contain triple mutant clones at the A/P boundary (red asterisks, Fig. 8c). Moreover, regardless of the number of orphan mutant clones present in the A compartment of the wing pouch (yellow asterisks, Fig. 8c), we generally observe a single triple mutant clone straddling the AP boundary (red asterisk, Fig. 8c; Supplementary Fig. 11). The observed mismatch in location and numbers of triple mutant and their sister clones suggests that the triple mutant cells do not mix well with surrounding wild-type cells, and instead appear to preferentially associate with each other and localize to the A/P boundary. In addition, the separation of triple mutant clones from their sister clones was only observed when large mosaic clones were induced very early during development, which indicates that the clonal segregation is a fairly slow process. Nevertheless, the predominant location of triple mutant clones at the A/P boundary suggests a preference of these clones for the location in the disc having the lowest level of Ihog/Boi protein.

As another approach to test whether pathway activation can reverse the segregation defects of *ihog/boi* double mutant clones we examined the effects of MARCM^75^-activated expression of a constitutively active form of Smo (*UAS-SmoGlu*)^76^ in large A clones near the A/P compartment boundary of the wing pouch. We found that pathway activation mediated by *UAS-SmoGlu* can rescue the abnormal segregation of *smo* mutant A clones (compare Supplementary Fig. 11A, B), but not of large *boi*; *ihog* mutant A clones near the A/P compartment boundary (compare Supplementary Fig. 11C, D–F). Most of the clones we examined were large, but we did note that smaller clones generated later during development are more likely to respect the A/P boundary, regardless of downstream pathway activation (see for example, Fig. 2b). The variation in segregation behavior related to clone size might explain why a previous study that examined mostly smaller clones failed to detect abnormal A/P segregation of clones lacking Ihog/Boi^56^. Although the MARCM system does not permit simultaneous visualization of twin spot sister clones, we did find that *boi*; *ihog*; *UAS-SmoGlu* clones are less frequently localized to the anterior compartment of the wing pouch, consistent with the abnormal segregation of sister *boi*; *ihog*; *pka-C1* mutant clones from *ihog* ^*+*^ clones noted above.

The inability of downstream pathway activation to rescue abnormal segregation behavior of *ihog*; *boi* clones at the A/P boundary (whether by *pka-C1* loss or MARCM-induced expression of *UAS-SmoGlu*), the dissociation of *boi*; *ihog* mutant clones from their *ihog* ^*+*^ sisters at a fivefold higher frequency than that previously reported for separation of wild-type twin spots, and the apparent migration and fusion of triple mutant clones at the A/P boundary together suggest that segregation defects of triple mutant cells are not due to a failure to transduce the Hh signal and that Ihog/Boi proteins likely contribute to A/P cell segregation in wing discs independently of their function as Hh receptor components (“Discussion”).

## Discussion

The Hh receptor component Ptc has two distinct functions: one in signaling via regulation of Smo and the other in Hh sequestration^40^. Previously, we demonstrated that Ihog/Boi interacts directly with Ptc, is required for presentation of Ptc on the cell surface, and that Ihog and Ptc are both required for high-affinity Hh binding^43^. This crucial role of Ihog/Boi in binding of Ptc to Hh suffices to explain why Ihog/Boi is indispensable for transduction of the Hh signal. With regard to Hh sequestration, restricting the range of Hh action in *Drosophila* wing imaginal discs requires depletion of Hh produced in P cells by the A cells immediately adjacent to the A/P boundary. These A cells not only must bind Hh but also eliminate it from the cell surface to prevent its further action upon dissociation^40, 41^. Here, we provide in vitro (Fig. 1) and in vivo (Fig. 2) evidence to show that Ihog/Boi is essential for Ptc-mediated Hh endocytosis, as well as for degradation of Ptc following Hh binding. Taken together, our evidence shows that the Ihog/Boi co-receptor, like Ptc itself, has dual roles in transducing and sequestering the Hh signal. Notably, Ihog/Boi activity in A cells: (i) enhances Hh binding and pathway activation, thus increasing the levels of Ptc expression that aid in adsorption of the Hh ligand^43^; and (ii) aids in Ptc-mediated Hh internalization and degradation upon binding, making the process of sequestration irreversible.

Ihog/Boi proteins contribute both to presentation of Ptc on the surface^43^ and to Hh-dependent Ptc degradation (Figs. 3–5). We note that Ihog itself is also internalized and degraded in a Hh-dependent manner, and that this activity is critically dependent upon co-expression of Ptc (Figs. 1, 3, 6, and 7). Ihog and Ptc thus interact physically, and Ihog and Ptc proteins are mutually required for surface presentation of the Hh receptor, for high-affinity binding^43^ and for Hh-dependent receptor internalization and degradation. These activities are summarized schematically in Fig. 8. On this basis and because of the absolute requirement for Ihog/Boi in all Hh biological responses and Hh sequestration, we consider Ihog to be an integral component of the *Drosophila* Hh receptor.

Despite a direct interaction between Ihog and Ptc and their joint contributions to formation, surface presentation, and ligand-dependent internalization of an active receptor, it is curious that these two pathway components have opposing effects on pathway activity. Ihog proteins thus are indispensable for positive response to Hh ligand, whereas Ptc in the absence of Hh ligand suffices to suppress Smo and pathway activity (Fig. 8). These two components are also distinctly regulated, with Ptc transcription increasing upon Hh stimulation, whereas Ihog protein levels decrease post-transcriptionally due to Hh-dependent and Ptc-dependent internalization and degradation; the distinct levels of Ihog/Boi protein across the wing disc thus is wholly predictable from the expression of Ptc and Hh, whether in their normal patterns or as altered by experimental manipulation.

With regard to cell segregation and lineage restriction at the A/P compartment boundary, the current view is that response to Hh signaling induces a change in adhesive properties of anterior cells abutting the compartment boundary, and that this difference leads to the segregation of A cells and P cells, and consequent creation of a lineage restriction. Differences in adhesiveness between cells could result from the presence of distinct types of cell adhesion molecules, or from different levels of the same cell adhesion molecule. However, despite extensive previous efforts to identify cell adhesion molecules that specify distinct A and P cell affinities, including systematic analyses of *Drosophila* cadherin-like proteins and screens for mutations that affect cell segregation^77, 78^, the identity of these hypothesized molecules remains unknown^77^.

Our finding that Ihog/Boi-expression can mediate aggregation of S2 cultured cells and that expression of Ihog/Ptc and Ihog/Hh in S2 cells to simulate A and P cell-surface character can cause segregation of these A- and P-like cells in vitro suggest that Ihog/Boi proteins may contribute to A/P cell segregation in vivo. The in vivo role of Ihog/Boi proteins in cell adhesion indeed is supported by our analysis of the *boi*; *ihog* mutant clones with pathway activation by *pka-C1* loss or MARCM-induced expression of *UAS-SmoGlu* originating near the A/P boundary. In these clones, the expression of downstream Hh pathway target genes induced by loss of *pka-C1* or by MARCM-induced expression of *UAS-SmoGlu* fails to rescue the cell segregation defects of *boi*; *ihog* mutant A cells, which remain segregated from adjacent A cells (Fig. 8b, c; Supplementary Fig. 11D–F). In addition, *boi*; *ihog*; *pka-C1* triple mutant clones originating in the A compartment, away from the A/P boundary, preferentially segregate away from their wild-type sister clones and appear to localize at the A/P boundary (Fig. 8c). This behavior contrasts sharply with that of wild-type clones^74^, and was not observed when either *ihog/boi* or *pka-C1* were mutated independently (Fig. 8a and Supplementary Fig. 9; see also refs. ^43, 69–72^).

If surface expression of Ihog/Boi specifies critical cell adhesion properties, why do *boi*; *ihog pka-C1* clones segregate away from their sister clones in the anterior whereas *boi*; *ihog* clones do not? We would speculate that this behavioral difference may be due to loss of *pka-C1* function, which enhances cell proliferation^69–72^. This proliferative difference could facilitate the initiation of movement for these triple mutant clones (Ganhui Lan, 2017, personal communication), whose eventual relocation to the A/P boundary may then be accounted for by the low level of Ihog/Boi adhesion proteins on cells at this location. Alternatively, it is possible that other cell adhesion molecules may contribute to cell segregation behavior, as posterior cells lacking Ihog/Boi function fail to invade the anterior compartment (Supplementary Fig. 12). Indeed, such a functionally overlapping role would be consistent with the suggestion of Vegh and Basler^77^ that multiple cell adhesion molecules may be involved in segregation of A/P compartment cells. Further, multiple factors in addition to cell adhesion differences may contribute to the boundary characteristics of clones, including internal forces mediated by cytoskeletal elements or by cell–cell adhesion, orientation of cell division and differential proliferation rates that generate patterns of mechanical tension^79–82^. It nevertheless remains striking that the Ihog/Boi proteins, shown here to mediate cell adhesion in vitro and to affect cell segregation under defined circumstances in vivo, show their greatest difference in expression between A cells and P cells at the compartment boundary of the wing imaginal disc.

## Methods

### Constructs

The *L*>*Ihog-GFP* and *L*>*Boi-GFP* transgenes were constructed by cloning Ihog/Boi-GFP fusion between the rp49 promoter and the hsp70 3′UTR in a pCasper vector^66^. Expression constructs of Ihog/Boi, Ptc variants used in *Drosophila* cell culture were fused in frame with a C-terminal HA tag, 3xMyc tag, GFP tag or mCherry tag, and cloned into the pAcSV plasmid. Constructs with Actin-Gal4 in pCasper vector and Rab5-GFP in the pUAST vector (a gift from M. Scott) were used to express Rab5 in S2R^+^ cells.

### Antibodies

Antibodies and dilutions used were: mouse anti-Ptc 1:50^63^, mouse anti-β-tubulin E7 1:5000 (DSHB, developed by M. Klymkowsky), mouse anti-HA 1:1000 (HA.11, Covance), rabbit anti-HhN 1:1000 (a gift from T. Tabata)^26^ for immunostaining, mouse anti-Myc (9E10, Santa Cruz), rabbit anti-Myc 1:1000 (A-14, Santa Cruz), rabbit anti GFP 1:1000 (Invitrogen, A-11122), rat anti-Ihog 1:200 for immunostaining and 1:1000 for immunoblotting^45^, HRP or fluorophore-conjugated secondary antibodies were from Jackson Immuno-Research Lab.

### Cell culture and transfection

*Drosophila* S2 and S2R*+* cells (DGRC) were cultured in *Drosophila* Schneider’s medium supplemented with 10% of fetal bovine serum and 1% penicillin–streptomycin–glutamine (Thermo Fisher) at 25 °C in a humidified incubator. Transfection was carried out using FuGENE 6 transfection reagent (Promega).

### Cell immunostaining

Forty-eight hours after transfection, cells were incubated 1 h with conditioned medium containing HhN protein or control conditioned medium. Cells were then washed twice with PBS, fixed in 4% formaldehyde (Ted Pella) in PBS, blocked by 1.5% normal goat serum (NGS) in PBS, incubated with primary antibody in PBS containing 1.5% NGS for overnight at 4° (to stain surface Ihog/Ptc/HhN), permeabilized by 0.3% Triton X-100/PBS for 15 min at room temperature, incubated with primary antibody in PBS containing 1.5% NGS and 0.3% Triton X-100 for 1 h at room temperature (to stain intracellular Ihog/Ptc/HhN), washed three times with 0.3% Triton X-100/PBS, incubated with secondary antibody and washed with 0.3% Triton X-100/PBS.

### Lysosome staining

S2R+ cells transfected with Ihog-YFP and Ptc-mCherry were incubated in HhN-conditioned medium or control medium for 1 h, live stained with 50 mM LysoTracker Deep Red (Thermo Fisher L12492) in media for 1 h at room temperature, washed with PBS for three times, and imaged with a confocal microscope.

### Cell-surface biotinylation

Cells were incubated in HhN-conditioned medium or control medium for 1 h, washed with PBS and incubated for 30 min in PBS containing 1 mg/ml Sulfo-NHS-LC-Biotin (Pierce, 21335). The reaction was quenched by washing the cells with 100 mM glycine in PBS three times. Cells were then lysed in 0.5% Digitonin (50 mM Tris-HCl pH = 7.5, 150 mM NaCl, and protease inhibitors) for 30 min. The lysate was clarified by centrifugation, and biotinylated protein was recovered by binding to Streptavidin-Sepharose beads (GE healthcare, 17-5113-01) for 2 h at room temperature. Beads were washed, and protein was recovered in SDS-PAGE sample buffer. Proteins samples were resolved by SDS-PAGE and transferred to PVDF membranes (Millipore) for western blot analysis. Uncropped western blot images can be found in Supplementary Fig. 13.

### RNAi

Primer pairs with each primer encoding a T7 promoter sequence and gene-specific sequence were used to amplify PCR sequence product from cDNAs to generate templates for in vitro dsRNA synthesis using T7 RNA polymerase (Ambion). See Supplementary Table 1 for a list of primers used. S2R+ cells were bathed in 15 μg/ml of dsRNA in serum-free medium for 1 h followed by addition of 10% FBS and transfection with 0.5 g of dsRNA using Effectene (Qiagen). Cells were incubated for an additional 2 days to allow protein turnover prior to treatment with control or HhN-containing medium.

### In situ hybridization

RNA probes were created from in vitro transcription of PCR products carrying the T7 RNA polymerase recognition sequence at one end and synthesized by using a digoxigenin (Dig)-labeling kit (Roche). Wing discs of L3 larvae were hybridized with probes overnight at 56 °C using standard procedures and visualized using anti-Dig-AP (1:1,000; Roche). Primers used for generating PCR templates are listed in Supplementary Table 2.

### *Drosophila* strains

Mutant and transgenic strains are listed in Supplementary Table 3.

### Genotype of larvae for generating mosaic clones

Wing imaginal disc clones were generated by FRT-FLP recombination. Fly embryos or first–second instar larvae were heat shocked for 1 h at 37 °C to produce large or smaller mosaic clones^40, 75^. The genotypes of larvae for generating mosaic clones are listed in Supplementary Table 4.

### Image collection and quantification of fluorescence intensity

To assess protein amounts in different subcellular domains, images used for comparison within an experiment were obtained with identical settings on a Zeiss Spinning Disc microscope and then used for quantification without any manipulation. A mask was constructed by manually outlining the cells in the image taken in the Rab5 or Lyso channel, for example. This mask was then applied to the image taken in the channel for the protein of interest, and the fluorescence was measured. Local background correction was performed by moving the mask to measure fluorescence at a representative nearby region and then subtracting this value from that of interest. All bars represent mean ± s.d. from 20 individual cells. Statistical analysis was performed using GraphPad Prism software.

To assess protein amounts in different wing imaginal discs, all wing discs from third instar larvae were dissected, fixed, stained in the presence or absence of detergent, and imaged in parallel using the same confocal microscope settings. To quantify Ihog, Ptc, and GFP-staining intensities, a rectangle was selected and centered at the AP boundary across both ventral and dorsal compartments. Average pixel intensity was determined using the Plot Profile function of ImageJ and plotted using GraphPad Prism software.

### Cell aggregation assay

S2 cells were transfected separately with plasmids expressing desired proteins. Forty-eight hours after transfection, S2 cells were washed with PBS and dissociated by 0.05% trypsin treatment for 5 min at 25 °C. The dissociated cells were resuspended in the medium with 10% fetal bovine serum, and differentially transfected cells were mixed in 1.5 ml eppendorf tubes. After rotating at room temperature for 4 h to allow aggregation to occur, cells were transferred into the wells of culture slides for live imaging by confocal microscopy.

### Statistical analyses

All data in column graphs are shown as mean values with s.d. Statistical analyses were performed with Mann–Whitney’s *U*-test as indicated in each figure legend. The sample sizes were set based on the variability of each assay and are listed in the figure legends. Independent experiments were performed as indicated to guarantee reproducibility of findings.

### Data availability

The authors declare that all data supporting the findings of this study are available within the article and its Supplementary Information files or from the corresponding authors upon reasonable request.

## Electronic supplementary material

Supplementary Information

Description of Additional Supplementary Files

Supplementary Movie 1

Supplementary Movie 2

Supplementary Movie 3

Supplementary Movie 4

Supplementary Movie 5

Supplementary Movie 6
