# Supplementary material for: Hedgehog mediated degradation of Ihog adhesion proteins modulates cell segregation in Drosophila wing imaginal discs
Source: Nat Commun. 2017 Nov 2;8:1275. doi: 10.1038/s41467-017-01364-z (PMC5668237; doi:10.1038/s41467-017-01364-z)
Supplement: Supplementary file 1 — Supplementary Information [file 41467_2017_1364_MOESM1_ESM.pdf]

## Supplementary Discussion

### Analysis of Camp et al. (2014)

Although many authors confirm the essential role of Ihog/Boi in mediating Hedgehog (Hh) signaling via Patched (Ptc) [1-4], and additional work confirms a role of Ihog/Boi in modulating Hh tissue movement [2,4-6], one group in particular failed to observe a role for Ihog/Boi in Hh sequestration, that is, the limitation of Hh signaling range across the A/P compartment boundary and into A cells [7]. Because our current work examines in depth the sequestration of the Hh protein at the A/P boundary and the mutual regulation of Ptc and Ihog/Boi in this process, we have undertaken a careful analysis of the work from this particular group. With regard to Hh sequestration, these authors' assertions are summarized as the "third conclusion" in the following passage from the discussion portion of their manuscript, here quoted in full:

"Our third conclusion is that Ihog and Boi, unlike Ptc, are completely dispensable for the sequestration and retention of Hh. We show that cells lacking Ihog and Boi can sequester and retain the Hh signal if the pathway is activated and Ptc is upregulated. They do so via physiological levels of endogenous Ptc induced either by pathway activation in $ptc^{S2}$ mutants or by expression of $Smo^{SD123}$. Incidentally, we also found that Hh sequestration was rescued in *boi;ihog* double mutant clones overexpressing $Ptc^{1130}$ (Fig. 7B), a dominant-negative that fully activates the Hh pathway and upregulates endogenous, wildtype Ptc. This third conclusion is not consistent with the view that Ihog and Boi aid in addressing Ptc to the cell surface and that, once there, they are required for Ptc to bind and sequester Hh (Zheng et al., 2010). This view is based primarily on Ptc and Ihog overexpression in cultured cells, and on an experiment that failed to

restore Hh sequestration to *boi;ihog* double mutant cells with mutation of cAMP-dependent protein kinase 1 (Pka-C1), which upregulates Ptc and other target genes because loss of Pka-C1 disinhibits the activity of the transcription factor Ci (Ohlmeyer and Kalderon, 1997; Chen et al., 1998; Price and Kalderon, 1999; Wang et al., 1999). It is unclear why Ptc upregulation in *Pka-C1* mutants was unable to rescue Hh sequestration in *boi;ihog* double mutants, whereas Ptc upregulation in our experiments was able to do so. In cells lacking Ihog and Boi, perhaps the level to which Ptc is upregulated in Pka-C1 mutants is inadequate."

**Ptc levels required for sequestration**

The major thrust of the assertions in Camp et al. [7] is to discount our experiment mutating the *pka* gene by saying that "perhaps the level to which Ptc is upregulated in Pka-C1 mutants is inadequate"; this assertion is demonstrably incorrect.

Chen and Struhl, who discovered Hh sequestration, made use of precisely this method (mutation of *pka*) to re-activate Ptc expression and restore Hh sequestration [8]. The fundamental observation of Chen and Struhl was that expression of Hh targets *ptc* and *dpp* occurs at abnormally long-range when Ptc induction by Hh in anterior (A) cells is prevented by mutation of *smo*. Chen and Struhl then showed that Hh sequestration and limitation of its signaling range was restored when Ptc protein expression was restored by mutation of *pka*. Mutation of *pka* restores Ptc expression to physiological levels because it mimics the induction of Ptc expression by Hh signaling (through downstream activation of Ci, the transcriptional effector of Hh signaling).

In our work, as seen in Supplementary Fig. 1A, we replicate this result from Chen and Struhl (1996) (i.e., we restore sequestration and prevent Hh response in distal cells anterior to a *smo* mutant clone by mutating *pka*). This rescue of sequestration clearly shows that the level of Ptc protein produced by *pka* is adequate for Hh sequestration, not "inadequate" as suggested by Camp et al. [7]. We then show that the sequestration by Ptc in a *pka* mutant clone is lost with the additional mutation of *boi; ihog* (Supplementary Fig. 1B). Our methodology in testing for rescue of sequestration is thus to cause expression of Ptc in the same way as Chen and Struhl, who first described the phenomenon.

The casual speculation that "perhaps the level to which Ptc is upregulated in Pka-C1 mutants is inadequate" [7] ignores the methods of the original demonstration of sequestration by Chen and Struhl, and fails to appreciate the importance of physiological levels of Ptc expression (see below).

**Physiological levels of Ptc expression?**

Camp et al. assert that "endogenous Ptc induced either by pathway activation in $ptc^{S2}$ mutants or by expression of $Smo^{SD123}$" is at "physiological levels". Their own data contradict this statement. For example, the Ptc protein level is much higher in *Actin-Gal4* driven *UAS-* $Smo^{SD123}$ clones than that in the surrounding cells at the A/P boundary of the same disc (note the white pseudo-color assignment, indicating saturating high levels of Ptc protein in their Supplementary Fig. 1B, C [7]). This high level of Ptc protein expression is not surprising, as the Gal4-dependent transgenic UAS promoter used will be extremely active when Gal4 is provided by actin-driven expression.

The Ptc level is also higher in $ptc^{S2}$ mutant clones than in other cells at the A/P boundary of the same disc (Fig. 6B and Supplementary Fig. 3D in Camp et al. [7]).

The criteria used to judge whether Hh sequestration occurs in general [7]are not consistent with the criteria used by Chen and Struhl in their original definition of sequestration [8]. Specifically, loss of Hh sequestration was defined as occurring when "(Hh) responses are induced ectopically in wild-type A cells positioned immediately anterior to mutant clones, often many cell diameters anterior to the normal A/P boundary" [8]. Thus, the Ptc level anterior to the mutant clone should be compared to the endogenous Ptc level outside the mutant clone, not to the level within the mutant clone (especially when $ptc^{S2}$ or $Smo^{SD123}$ induced Ptc levels are abnormally high, see above). Furthermore, given the large differences in expression levels of endogenous Ptc in different discs (e.g. Supplementary Fig.1 in Camp et al. [7]), it is also inappropriate to compare Ptc levels anterior to the mutant clones between different discs without normalization (Fig. 3 and Supplementary Fig. 1 in Camp et al.[7]).

**$Ptc^{1130}$**

Camp et al. [7] state that "we also found that Hh sequestration was rescued in *boi;ihog* double mutant clones overexpressing $Ptc^{1130}$ (Fig. 7B), a dominant-negative that fully activates the Hh pathway and upregulates endogenous, wild-type Ptc."

Data supporting this claim are absent. Wing discs carrying $Ptch^{1130}$ clones are shown in Fig. 7B[7], but were only stained to visualize expression of Ci155. The levels of Ci155 are known to be lower in cells close to the source of Hh as compared to those in more distal regions of the Hh-

responsive domain (the most active form of Ci155 is less stable; see [9]). Reduced Ci155 staining thus is not a reasonable basis for concluding that Hh sequestration occurs, especially as no staining for Ptc or Hh are presented. Camp et al. [7] also entirely ignored our experiments clearly showing that ectopic expression of $Ptch^{1130}$ failed to rescue Hh sequestration in *ihog;boi* clones, based on anti-Hh staining (Fig 6 in Zheng et al. [4]). Selective disregard of inconvenient data is also suggested by their assertion that our view "is based primarily on Ptc and Ihog overexpression in cultured cells". To the contrary, our view is based on multiple experiments in imaginal discs (Figs. 2,3, 6 in Zheng et al. [4]) and in cultured cells (Fig 4, 7 in Zheng et al. [4]).

**Clonal analysis with *boi* and *ihog* alleles**

In the experiments with a constitutively activated form of Smo ($Smo^{SD123}$) [10] Camp et al. used various uncharacterized *boi* and *ihog* alleles that might retain the Hh-binding FNIII domain (Fn1) [11] and thus some residual function [3]. In addition, some mutant clones used in their statistical analysis were triangularly-shaped, complicating interpretation regarding Hh sequestration.

**Additional considerations**

Camp et al. present no biochemical data of their own and do not contest our biochemical and crystallographic structural data clearly demonstrating that Hh interacts specifically with Ihog and with Ptc. In fact, their article closes as follows [7]: "Together, these results suggest that the primary role of Ihog and Boi in Hh signaling involves the ability of their ectodomains to form favorable protein complexes with Ptc or Hh, or with both simultaneously… we surmise that it is through

these multimolecular complexes that Ihog and Boi allow Hh to inhibit Ptc and thereby relieve its suppression of Smo and the Hh signaling cascade.”

Given their acceptance of a physical interaction between Hh and the Ihog/Boi:Ptc co-receptor, we find it curious that Camp et al. did not consider the reasonable question of how is it possible that such interactions would not contribute to Hh ligand sequestration?

In addition, we demonstrate in the current manuscript that Ihog/Boi protein levels are normally modulated downward in a stripe of cells at the A/P compartment boundary (Fig. 5) or at other locations in the imaginal disc (Fig. 6) in a manner that depends critically on Hh and Ptc expression. It is difficult to imagine how this discontinuity in Ihog/Boi levels could be achieved without participation of Ihog/Boi in ligand-induced receptor degradation.

Based on a careful analysis of the data in Camp et al. [7] (see above), and on consideration of our prior data [4] as well as the new data in Supplementary Fig. 1, our conclusion remains that Ihog/Boi expression is required for the normal sequestration of Hh to limit its range of action, as previously stated [4]. In the main text of this article, we dissect the mechanism underlying Ihog/Boi-Ptc dependent Hh sequestration and its consequences for modulation of Ihog/Boi at the A/P compartment boundary.

**Supplementary Figures**

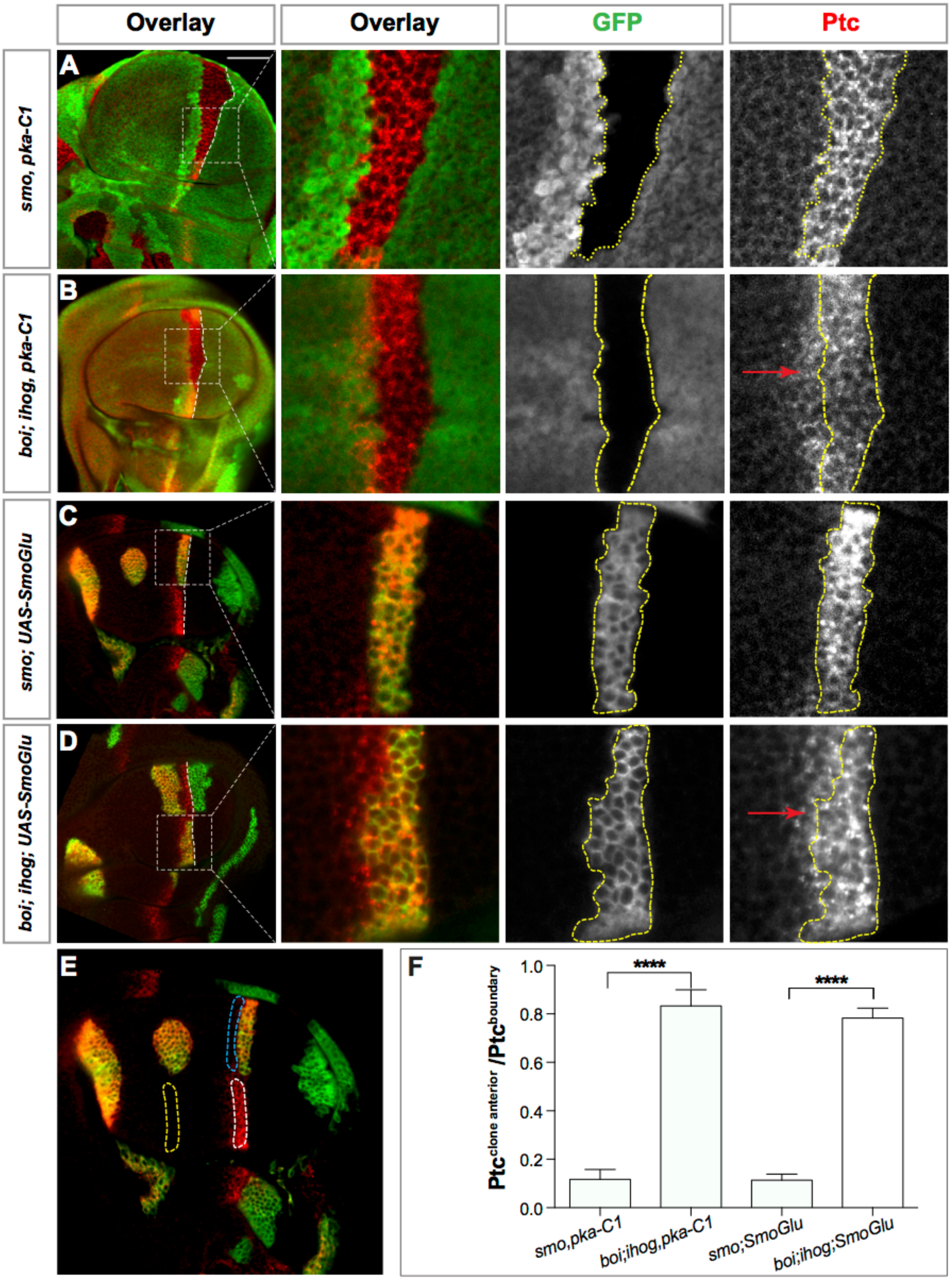

**Supplementary Figure 1. Up-regulation of Ptc is not sufficient to sequester Hh in the absence of Ihog/Boi.** (A-D) Each set of panels shows a wing imaginal disc immunostained for GFP (green) and Ptc (red). (A, C) *smo, pka-C1* mutant clones (panel A, GFP negative) or *smo* mutant MARCM clones expressing *UAS-SmoGlu* (panel C, mCD8GFP positive) originating in the A compartment autonomously express Ptc and sequester the Hh signal. Note that high-level Ptc expression is restricted to cells within either the *smo, pka-C1* mutant clone or the *UAS-SmoGlu*-expressing *smo* mutant MARCM clone (yellow lines). (B, D) *boi; ihog, pka-C1* mutant clones (panel B, GFP negative) or *boi; ihog* mutant MARCM clones expressing *UAS-SmoGlu* (panel D, mCD8GFP positive) originating in the A compartment autonomously express Ptc, but still fail to sequester the Hh signal. Note expression of Ptc in thin strips of cells (red arrows) immediately anterior to the border (yellow lines) of the *boi; ihog, pka-C1* mutant clone or *UAS-SmoGlu* expressing *boi; ihog* mutant MARCM clone, indicating that elevated Ptc expression alone is not sufficient to sequester the Hh signal, and that Ihog/Boi is absolutely required to limit the range of Hh activity. White lines indicate the border of Hh-secreting posterior cells. Scale bar, 50 µm. (E) Quantification example. Average Ptc intensity in normal boundary A cells (within white outline), average Ptc intensity anterior to the clone (within blue outline, consists of the first two rows of cells) and average Ptc intensity in A cells far from AP boundary (yellow outline) were measured in each discs using ImageJ. (F) $Ptc^{clone\ anterior}/Ptc^{boundary}$ is mean fluorescence intensity of Ptc in cells anterior to the mutant clone ($Ptc^{clone\ anterior} = Ptc^{blue} - Ptc^{yellow}$) normalized by Ptc intensity in normal boundary cells from the same wing disc ($Ptc^{boundary} = Ptc^{white} - Ptc^{yellow}$). Each bar shows the mean ± s.d. from 5 clones from 5 different discs, and representative images are shown in (A-D). Two-tailed unpaired Mann–Whitney *U*-test was used for statistical analysis. ns, not significant. ****$P < 0.0001$.

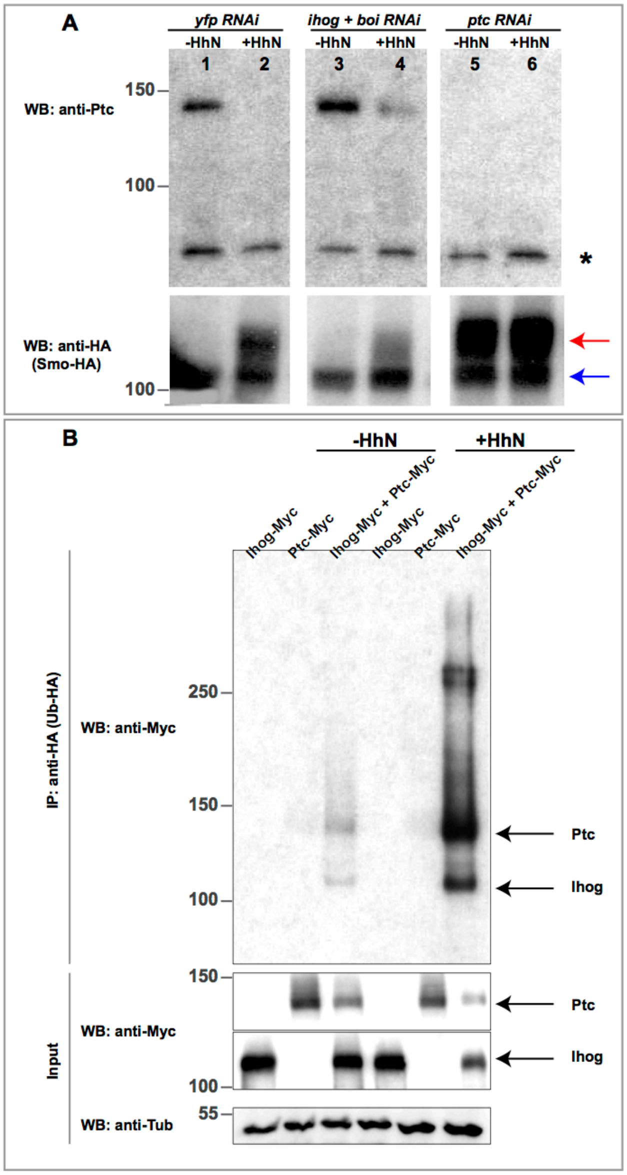


**Supplementary Figure 2. Ihog/Boi Mediates Hh-Dependent Degradation of Ptc *in vitro*** (A) S2R+ cells were transfected with HA-tagged Smo and the indicated dsRNA. Cells were lysed after 4 hours treatment with either HhN-containing or control conditioned medium and proteins were detected by immunoblotting for endogenous Ptc (anti-Ptc) or transfected Smo (anti-HA). HhN stimulation caused degradation of endogenous Ptc protein (lane 2), but Ptc degradation was reduced when cells were transfected with dsRNA against *ihog* and *boi* (compare lane 4 to lane 2; the asterisk denotes a background band unaltered by HhN stimulation that serves as an internal control). In the lower panel, either HhN stimulation or dsRNA against *ptc* caused accumulation of phosphorylated Smo (red arrow, lanes 2, 5 and 6), whereas dsRNA against *boi* and *ihog* dramatically reduced the Hh-induced accumulation of phosphorylated Smo (lane 4). (B) S2R+ cells were transfected with HA-tagged Ub together with Myc tagged Ihog, Ptc or both. After 1hr incubation with HhN containing medium and MG132, cell lysate was collected. Cell lysates were precipitated by anti-HA antibody and ubiquitinated Ihog or Ptc were detected by anti-Myc antibody.

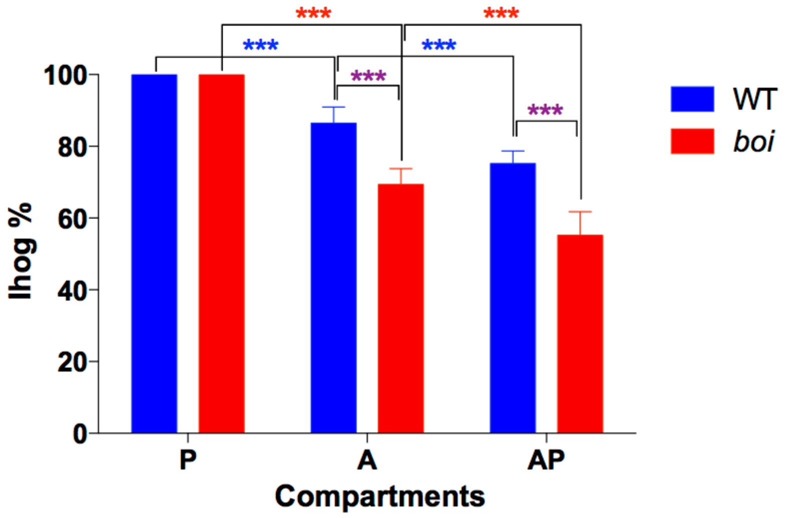


**Supplementary Figure 3. Quantification of Ihog protein levels across the wing disc.** Quantification of Ihog intensity in different compartments determined by Ptc staining both in wing discs from the wild-type or *boi* mutant larvae. Ihog intensity at different locations is normalized to that of the P compartment in the same wing disc. Each bar shows the mean ± s.d. of normalized Ihog intensity from n=7 wing discs, and representative images are shown in Fig 5A, B. Two-tailed unpaired Mann–Whitney *U*-test was used for statistical analysis. ns, not significant. ***P < 0.001.

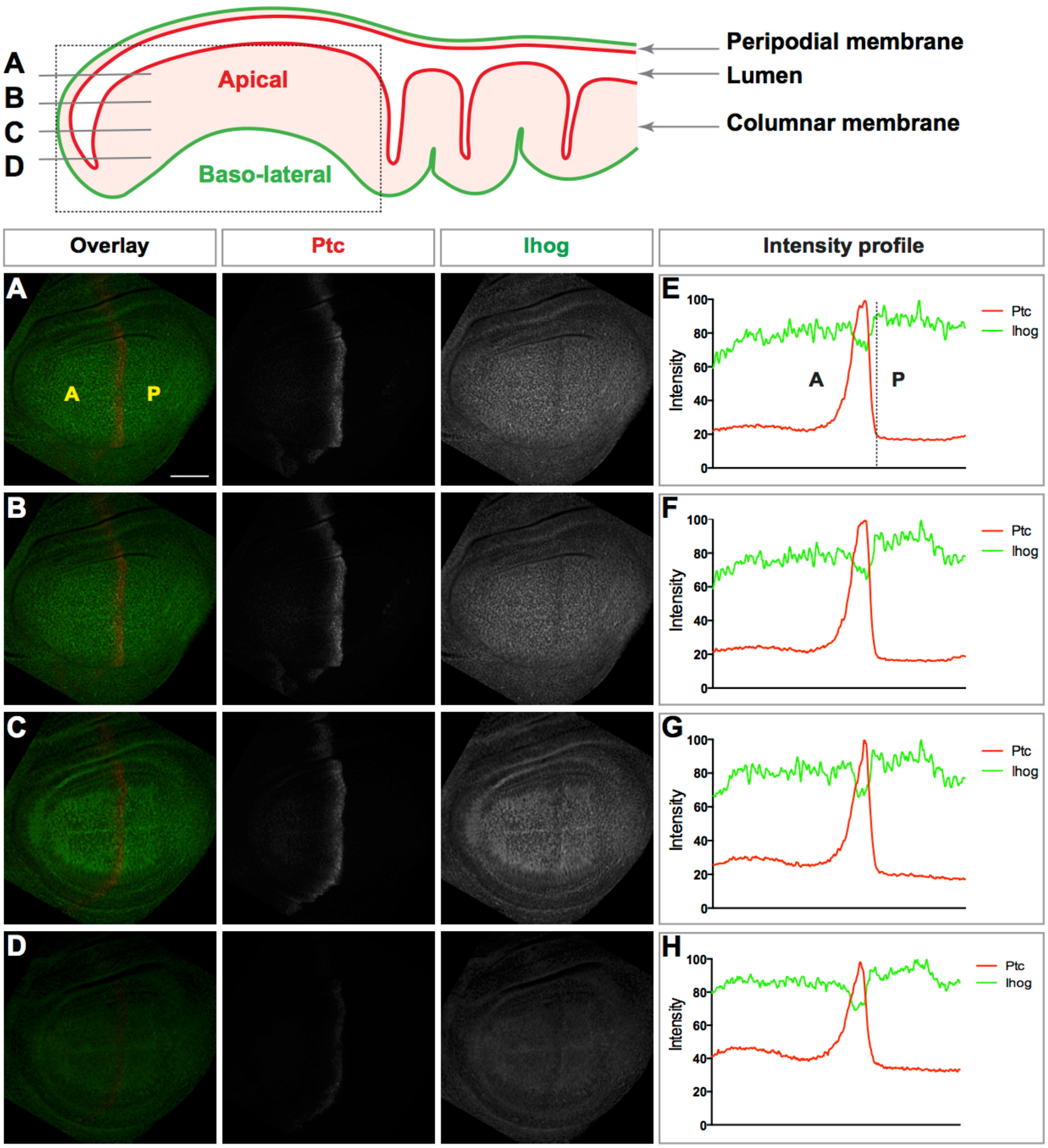


**Supplementary Figure 4. Ihog expression across the columnar epithelium of the wild-type wing discs.** (A-D) Wing imaginal discs from wild-type larvae were immunostained for Ihog (green) and Ptc (red). Lower Ihog levels were detected in the A compartment, and lowest levels occur in the Ptc-expressing cells adjacent to the A/P compartment boundary. Panels A-D show four different focal planes from the apical to the basal surface through the columnar epithelium of the wing discs. The relative position of A-D is indicated in the schemes of a cross section view of third instar wing discs (Top). See Supplementary Figure 7A for maximum intensity projection. See also Supplementary Movies 1-3. Scale bar, 50 µm. (E-H) Plotted normalized pixel intensities of Ptc and Ihog or GFP as a function of A/P position.

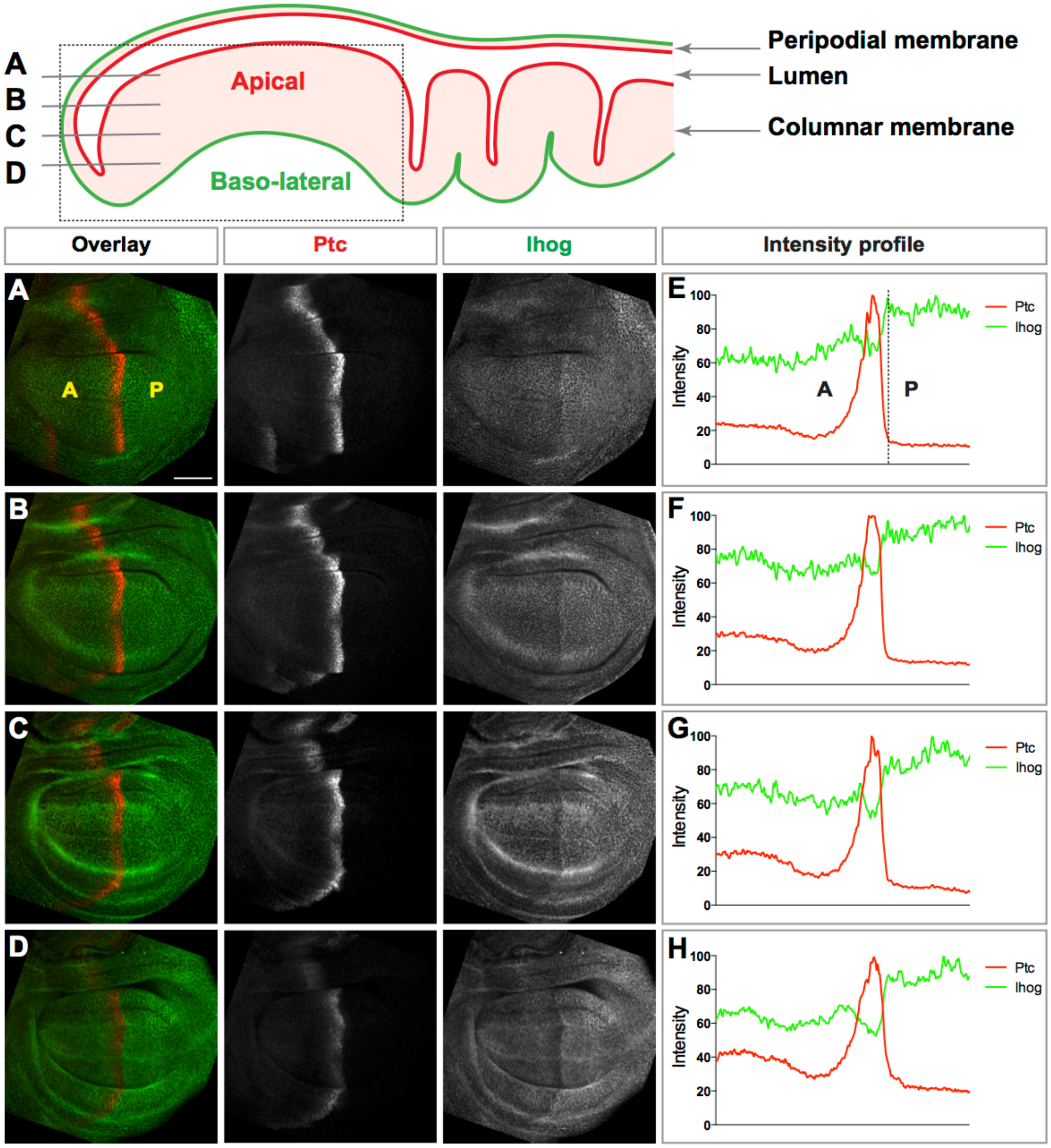


**Supplementary Figure 5. Ihog expression across the columnar epithelium of *boi* mutant wing discs.** (A-D) Wing imaginal discs from *boi* mutant larvae were immunostained for Ihog (green) and Ptc (red). Lower Ihog levels were detected in the A compartment, and lowest levels occur in the Ptc-expressing cells adjacent to the A/P compartment boundary. Panels A-D show four different focal planes from the apical to the basal surface through the columnar epithelium of the wing discs. The relative position of A-D is indicated in the schemes of a cross section view of third instar wing discs (Top). See also Supplementary Moviess 4-6. Scale bar, 50 µm. (E-H) Plotted normalized pixel intensities of Ptc and Ihog or GFP as a function of A/P position.

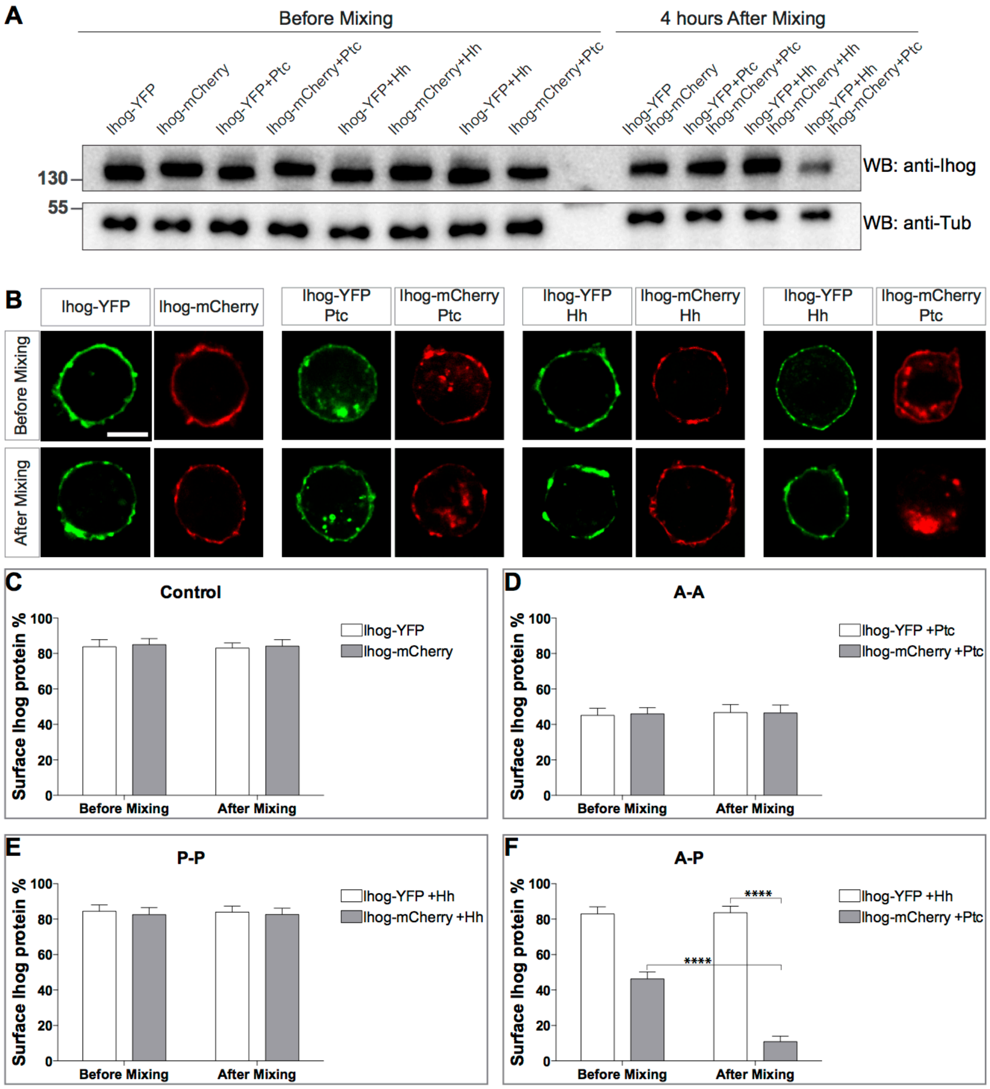


**Supplementary Figure 6. Quantification of Ihog protein level in S2 cells analyzed in the cell-mixing assay.** As described in Figure 7, S2 cells were transfected separately with plasmids expressing Ihog-YFP/mCherry, Ihog-YFP/mCherry and Hh, or Ihog-YFP/mCherry and Ptc. Cells were dissociated by trypsin treatment and then mixed 4 hr to allow aggregation to occur. (A) Aliquots of the same cells analyzed in the cell mixing assay were lysed before or 4 hours after cell mixing. The total Ihog protein levels (anti-Ihog) were detected by immunoblotting. (B) Aliquots of the same cells analyzed in cell mixing assay were also dissociated and imaged by confocal microscope. Subcellular localization of transfected Ihog proteins was visualized based on their mCherry (red) and YFP (green) tags. Scale bar, 5 μm. (C-F) Quantification of Ihog protein intensity on the cell surface. Surface Ihog protein % = Ihog surface intensity / (Ihog surface intensity + Ihog internal intensity) %. Each bar shows the mean ± s.d. from n=20 cells, and representative images are shown in (B). Two-tailed unpaired Mann–Whitney *U*-test was used for statistical analysis. ns, not significant. ****P < 0.0001.

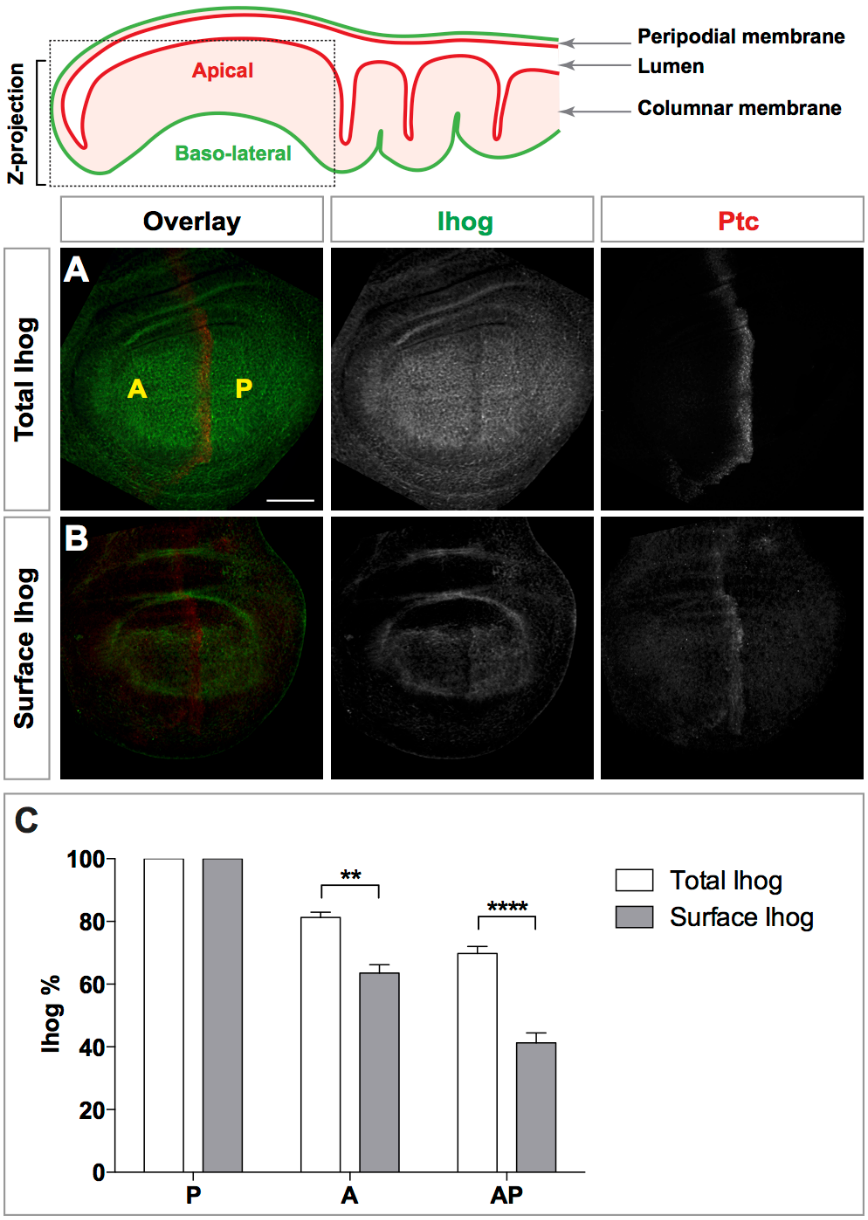


**Supplementary Figure 7. Surface Ihog expression in the columnar epithelium of the wild-type wing discs.** (A-B) Maximum intensity projections of x-y sections across apical to basal surface of the columnar epithelium from wild-type wing discs. The wing discs were immunostained using antibodies against the extracellular domain for Ihog (green) and Ptc (red), both in the presence (A) and in the absence (B) of detergent. Note lower Ihog levels were detected in the A compartment, and lowest levels occur in the Ptc-expressing cells adjacent to the A/P compartment boundary. Scale bar, 50 µm. (C) Quantification of total Ihog protein (A) and surface Ihog protein level (B) across the wing discs. Ihog intensity at different locations is normalized by that of P compartment of the same wing disc. Each bar shows the mean ± s.d. of normalized Ihog intensity from n=10 wing discs, and representative images are shown in (A, B). Two-tailed unpaired Mann–Whitney *U*-test was used for statistical analysis. ns, not significant. $^{**}P < 0.01$. $^{****}P < 0.0001$.

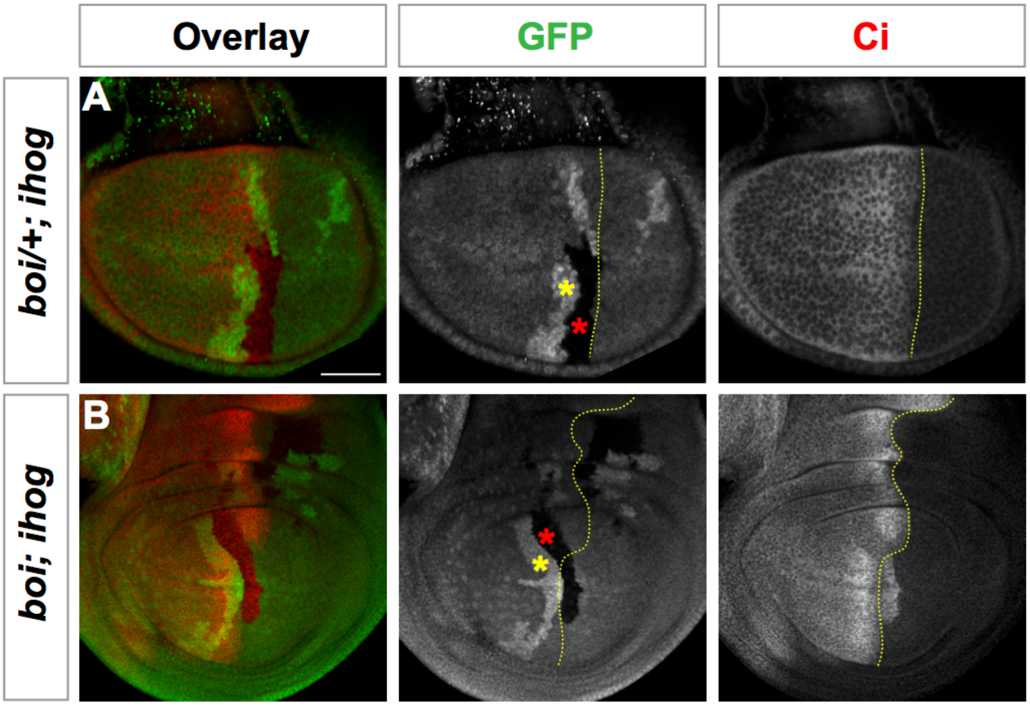


**Supplementary Figure 8. *ihog/boi* disrupts A/P cell segregation at the compartment boundary.** (A-B) Each set of panels shows a wing imaginal disc immunostained for GFP (green), Ci (red - marking cells of A compartment origin). Homozygous *ihog* mutant clones (red asterisk) are marked by loss of GFP expression, their *ihog*$^+$/*ihog*$^+$ sister clones (yellow asterisk) are marked by elevated levels of GFP expression. As indicated by expression of Ci, the approximate location of the compartment boundary is indicated by a dotted yellow line. (A) Homozygous *ihog*, heterozygous *boi* mutant clones (*boi/+; ihog*) at the compartment boundary within the wing pouch form a straight and smooth border with P compartment cells. (B) A large homozygous *boi; ihog* mutant clone (*boi; ihog*) within the wing pouch and originating in the A compartment (as judged by expression of Ci, and by position of the *ihog*$^+$/*ihog*$^+$ sister clone) is partly located in territory normally occupied by P compartment cells. Scale bar, 50 µm.

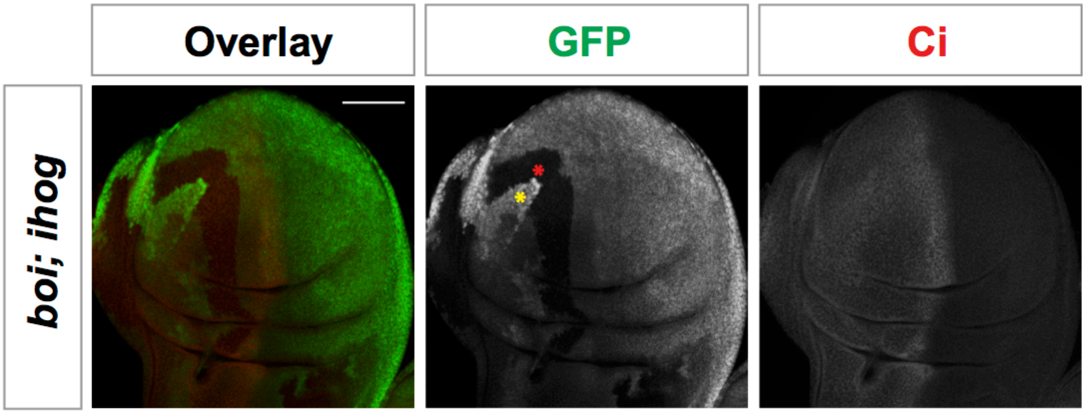


**Supplementary Figure 9. Clones lacking Ihog/Boi in the A compartment away from the A/P boundary.** The wing imaginal discs from third instar larvae are immunostained for GFP (green) and Ci (red). The mutant clones lacking both Ihog and Boi are marked by the absence of GFP expression (red asterisks), and the wild-type sister clones composed of cells that have two copies of GFP and thus are marked by the elevated level of GFP expression (yellow asterisks). Note the sister clones are located next to each other. Scale bar, 50 μm.

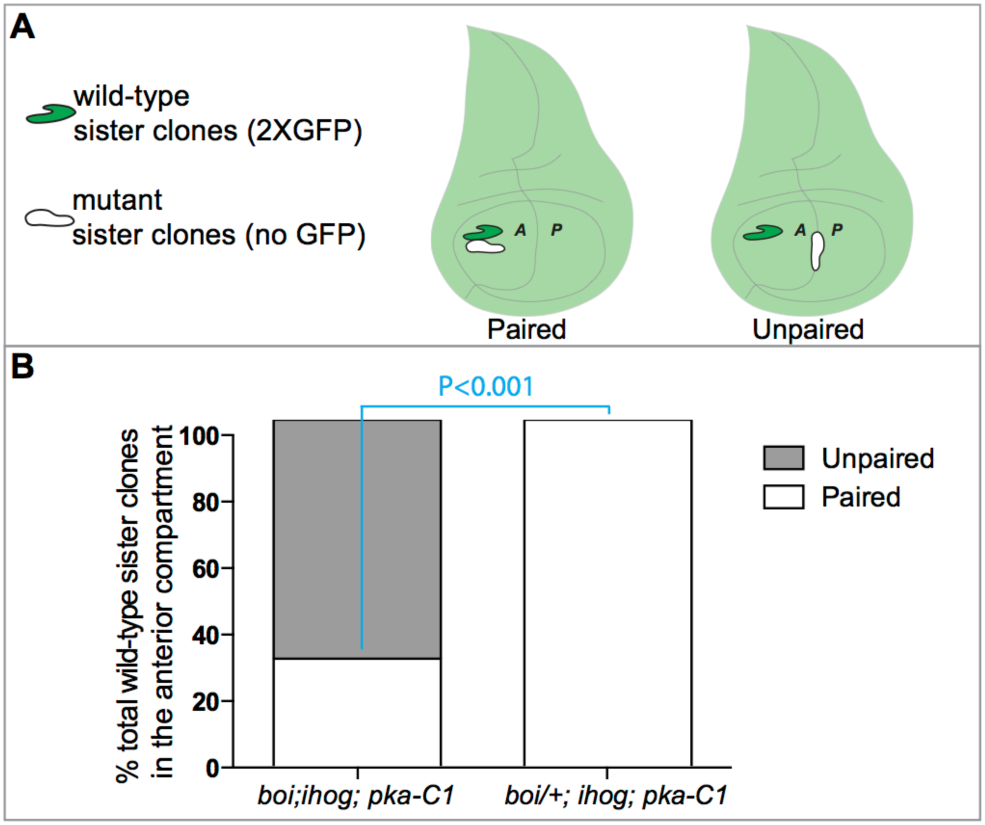


**Supplementary Figure 10. Quantification of the behavior of large clones lacking Ihog/Boi and Pka-C1.** As described in Figure 8, wing imaginal disc carrying large mutant clones were immunostained for GFP and Ci. The mutant sister clones are marked by the absence of GFP expression, and the wild-type sister clones composed of cells that have two copies of GFP and thus are marked by elevated GFP expression. (A) Schematic diagram to define paired and unpaired sister clones in the wing imaginal discs. (B) Quantification of paired and unpaired wild-type sister clones located in the anterior compartment of wing imaginal discs. Whereas all sister clones with *ihog*$^{+}$ function (12/12) remained paired, 9/13 *ihog*$^{+}$ clones expected to have sisters lacking *boi; ihog* function were unpaired. Two-sided Fisher's exact test was used for statistical analysis. P < 0.001.

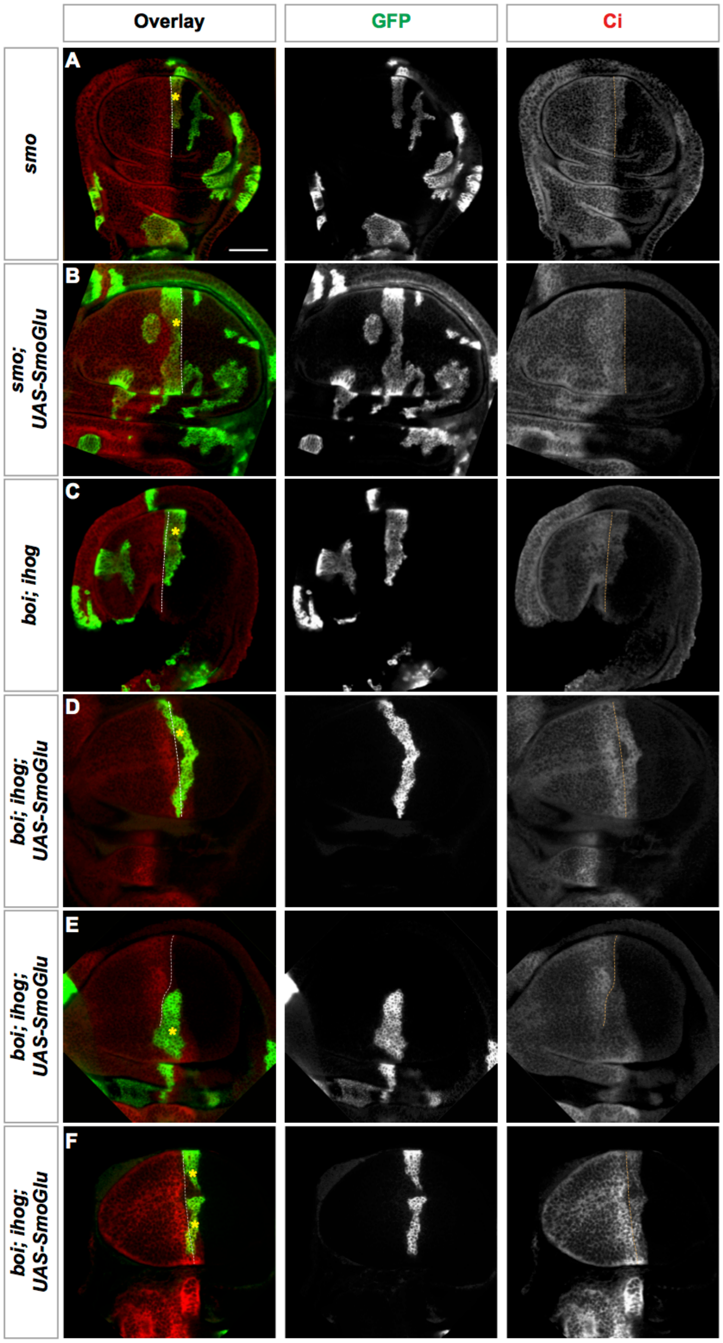


**Supplementary Figure 11. The behavior of large *boi; ihog* mutant A clones expressing activated Smo.** Each set of panels shows a wing imaginal disc immunostained for GFP (green) and Ci (red). The dashed line indicates predicted AP compartment boundary based on Ci expression pattern. (A, B) *smo* or (C-F) *boi; ihog* mutant MARCM clones are indicated by expression of mCD8GFP. (B, D-F) Mutant MARCM clones with indicated genotypes also express *UAS-SmoGlu*. Ectopic SmoGlu expression restricts large *smo* mutant clones (B), but not large *boi; ihog* mutant clones located at the A/P boundary (D-F), to the A compartment of wing discs. Scale bar, 50 μm.

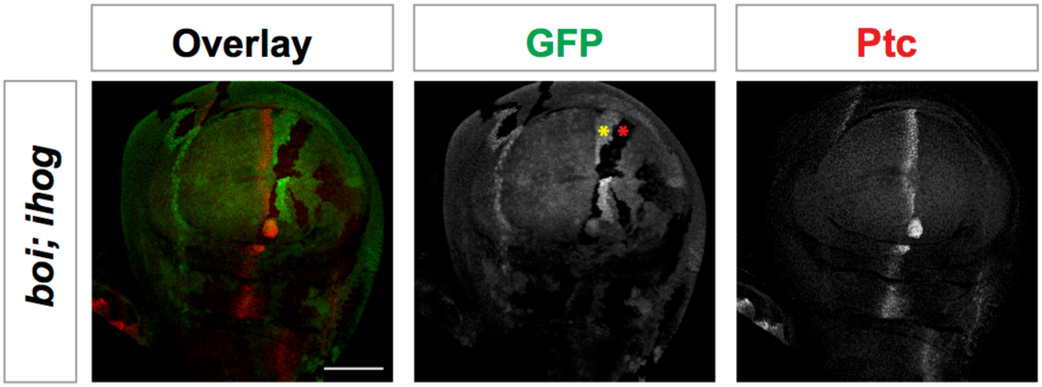


**Supplementary Figure 12. P Clones lacking Ihog/Boi form a rough boundary with surrounding cells.** The wing imaginal discs from third instar larvae are immunostained for GFP (green) and Ci (red). The mutant clones lacking both Ihog and Boi are marked by the absence of GFP expression (red asterisks), and the wild-type sister clones composed of cells that have two copies of GFP and thus are marked by elevated GFP expression (yellow asterisks). Note the mutant clones form a rough boundary with surrounding cells, and are located next to their wild-type sister clone. Scale bar, 50 µm.

Figure 1J

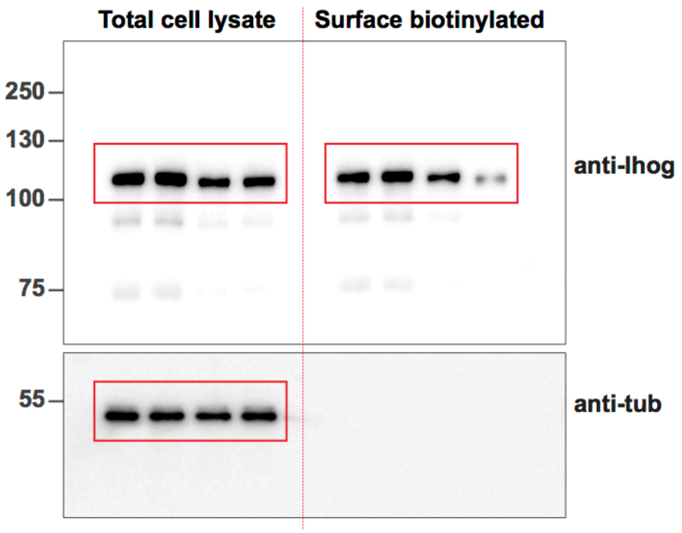


Supplementary Figure 6A

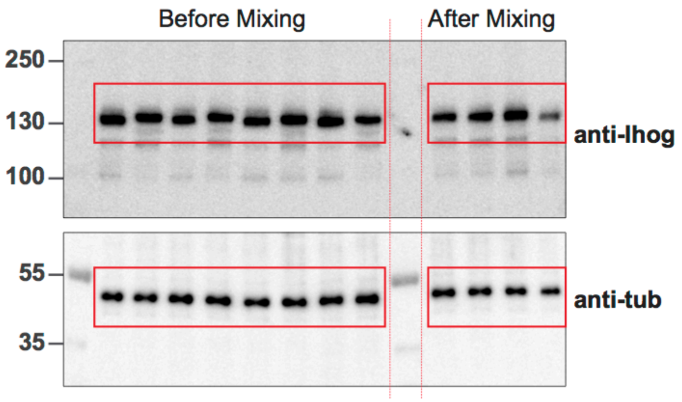


**Supplementary Figure 13. Uncropped western blot images.**

| Gene | Primer pairs |
|---|---|
| *yfp* | TAATACGACTCACTATAGGGatggtgagcaagggcgag |
| | TAATACGACTCACTATAGGGgaagttcaccttgatgcc |
| *ptc* | TAATACGACTCACTATAGGGatggaccgcgacagcctccca |
| | TAATACGACTCACTATAGGGgaggtggcgcaggatctgctc |
| *ihog* | TAATACGACTCACTATAGGGgcgggctatgtccaatgctttg |
| | TAATACGACTCACTATAGGGgcaccacagcggtttcagagtattc |
| *boi* | TAATACGACTCACTATAGGGgttgggcgacggaaaaaatc |
| | TAATACGACTCACTATAGGGtccagagttgttcttatcggatgc |

*Uppercase letters indicate the T7 promoter sequence.

**Supplementary Table 1. Primers used for dsRNA synthesis**

| PCR templates | Primers |
|---|---|
| sense probe (600bp) for *ptc* | TAATACGACTCACTATAGGGatggaccgcgacagcctccca and gaggtggcgcaggatctgct |
| anti-sense probe (600bp) for ptc | atggaccgcgacagcctccca and TAATACGACTCACTATAGGGgaggtggcgcaggatctgctc |
| sense probe (547bp) for *ihog* | TAATACGACTCACTATAGGGgcgggctatgtccaatgctttg and gcaccacagcggtttcagagtattc |
| anti-sense probe (547bp) for *ihog* | gcgggctatgtccaatgctttg and TAATACGACTCACTATAGGGgcaccacagcggtttcagagtattc |
| sense probe (594bp) for *boi* | TAATACGACTCACTATAGGGcgagcgagggaaacatattg and ctggcagattcgacttggac |
| anti-sense probe (594bp) for *boi* | cgagcgagggaaacatattg and TAATACGACTCACTATAGGGctggcagattcgacttggac |

*Uppercase letters indicate the T7 RNA polymerase recognition sequence.

**Supplementary Table 2. Primers used for generating *in situ* probes**

| *Drosophila* strains | Reference |
|---|---|
| *pka-C1*$^{B3}$ | Lane & Kalderon, *Genes & Development* 7, 1229-1243 (1993) [12]. |
| *Act5C>CD2>Gal4* | Pignoni & Zipursky, *Development* 124, 271-278 (1997)[13]. |
| *Actin-Gal4* | Ito et al., Development 124, 761-771 (1997)[14]. |
| *hs-FLP* | Golic & Lindquist, Cell 59, 499-509 (1989)[15]. |
| *tubP-Gal80* | Lee & Luo, *Neuron* 22, 451-461 (1999)[16]. |
| *tubP-Ptc* (*Tubα1P-Ptc-Tubα1 3'UTR)* | Chen & Struhl, Cell 87, 553-563 (1996)[8]. |
| *UAS-mCD8GFP* | Lee & Luo, *Neuron* 22, 451-461 (1999)[16]. |
| *UAS-SmoGlu* | Zhang et al., PNAS 101, 17900-17907, (2004)[17]. |
| *UAS-Hh* | Azpiazu et al., *Genes & Development* 10, 3183-3194 (1996)[18]. |
| *UAS-Ptc* | Johnson et al., *Mol Cell* 6, 467-478 (2000)[19]. |
| *smo*$^{3}$ | Chen & Struhl, Cell 87, 553-563 (1996)[8]. |
| *boi/ihog* | Zheng et al., *Genes Dev* 24, 57-71(2010)[4]. |

**Supplementary Table 3. Mutant and transgenic *Drosophila* strains**

*Wing Disc MARCM Clones*

- Positively marked *ihog* mutant clones in a *boi* heterozygous background: *y w boi hs-FLP UAS-mCD8GFP/X; ihog FRT40A/tubP-GAL80 FRT40A; ActP-Gal4/Tm6B*
- Positively marked *ihog* mutant clones in a *boi* homozygous or hemizygous background: *y w boi hs-FLP UAS-mCD8GFP/y w boi or Y; ihog FRT40A/tubP-GAL80 FRT40A; ActP-Gal4/Tm6B*
- Positively marked *ihog* mutant clones in a *boi* homozygous or hemizygous background simultaneously expressing *UAS-Ptc or UAS-SmoGlu*: *y w boi hs-FLP UAS-mCD8GFP/y w boi or Y; ihog FRT40A/tubP-GAL80 FRT40A; ActP-Gal4/UAS-Ptc (or UAS-SmoGlu)*
- Positively marked $smo^3$ mutant clones simultaneously expressing *UAS-SmoGlu:* $smo^3$ *FRT40A/tubP-GAL80 FRT40A; ActP-Gal4/UAS-SmoGlu.*

*Wing Disc MARCM Clones in the Background of tubP-Ptc*

- Positively marked *ihog* mutant clones in a *boi* heterozygous background: *y w boi hs-FLP UAS-mCD8GFP/X; ihog FRT40A/tubP-GAL80 FRT40A; ActP-Gal4/ tubP-Ptc*
- Positively marked *ihog* mutant clones in a *boi* hemizygous background: *y w boi hs-FLP UAS-mCD8GFP/Y; ihog FRT40A/tubP-GAL80 FRT40A; ActP-Gal4/ tubP-Ptc*

*Flip-out Clones for Activation of Hh or Ptc expression*

- Ptc: *hs-FLP UAS-mCD8GFP/X; Act5C>CD2>Gal4/+; UAS-Ptc/+*
- Hh: *hs-FLP UAS-mCD8GFP/X; Act5C>CD2>Gal4/UAS-Hh*

*Wing Disc Clones Marked by Absence of GFP*

- *ihog and* $pka\text{-}C1^{B3}$ double mutant clones in a *boi* homozygous or hemizygous background: *y w boi hs-FLP/y w boi or Y; ihog* $pka\text{-}C1^{B3}$ *FRT40A/ubi-nGFP FRT40A*
- $smo^3$ *and* $pka\text{-}C1^{B3}$ double mutant clones: $smo^3$ $pka\text{-}C1^{B3}$ *FRT40A/ ubi-nGFP FRT40A*

**Supplementary Table 4. Genotype of larvae for generating mosaic clones**

## Supplementary References:


1 Chou, Y. H., Zheng, X., Beachy, P. A. & Luo, L. Patterning axon targeting of olfactory receptor neurons by coupled hedgehog signaling at two distinct steps. *Cell* **142**, 954-966, doi:10.1016/j.cell.2010.08.015 (2010).

2 Yan, D. *et al.* The cell-surface proteins Dally-like and Ihog differentially regulate Hedgehog signaling strength and range during development. *Development* **137**, 2033-2044, doi:10.1242/dev.045740 (2010).

3 Camp, D., Currie, K., Labbe, A., van Meyel, D. J. & Charron, F. Ihog and Boi are essential for Hedgehog signaling in Drosophila. *Neural development* **5**, 28, doi:10.1186/1749-8104-5-28 (2010).

4 Zheng, X., Mann, R. K., Sever, N. & Beachy, P. A. Genetic and biochemical definition of the Hedgehog receptor. *Genes Dev* **24**, 57-71, doi:10.1101/gad.1870310 (2010).

5 Hartman, T. R. *et al.* Drosophila Boi limits Hedgehog levels to suppress follicle stem cell proliferation. *J Cell Biol* **191**, 943-952, doi:10.1083/jcb.201007142 (2010).

6 Hartman, T. R., Strochlic, T. I., Ji, Y., Zinshteyn, D. & O'Reilly, A. M. Diet controls Drosophila follicle stem cell proliferation via Hedgehog sequestration and release. *J Cell Biol* **201**, 741-757, doi:10.1083/jcb.201212094 (2013).

7 Camp, D. *et al.* Ihog and Boi elicit Hh signaling via Ptc but do not aid Ptc in sequestering the Hh ligand. *Development* **141**, 3879-3888, doi:10.1242/dev.103564 (2014).

8 Chen, Y. & Struhl, G. Dual roles for patched in sequestering and transducing Hedgehog. *Cell* **87**, 553-563, doi:10.1016/S0092-8674(00)81374-4 (1996).

9 Ohlmeyer, J. T. & Kalderon, D. Hedgehog stimulates maturation of Cubitus interruptus into a labile transcriptional activator. *Nature* **396**, 749-753, doi:10.1038/25533 (1998).

10 Jia, J., Tong, C., Wang, B., Luo, L. & Jiang, J. Hedgehog signalling activity of Smoothened requires phosphorylation by protein kinase A and casein kinase I. *Nature* **432**, 1045-1050, doi:10.1038/nature03179 (2004).

11 McLellan, J. S. *et al.* Structure of a heparin-dependent complex of Hedgehog and Ihog. *Proc Natl Acad Sci U S A* **103**, 17208-17213, doi:10.1073/pnas.0606738103 (2006).

12 Lane, M. E. & Kalderon, D. Genetic investigation of cAMP-dependent protein kinase function in Drosophila development. *Genes Dev* **7**, 1229-1243 (1993).

13 Pignoni, F. & Zipursky, S. L. Induction of Drosophila eye development by decapentaplegic. *Development* **124**, 271-278 (1997).

14 Ito, K., Awano, W., Suzuki, K., Hiromi, Y. & Yamamoto, D. The Drosophila mushroom body is a quadruple structure of clonal units each of which contains a virtually identical set of neurones and glial cells. *Development* **124**, 761-771 (1997).

15 Golic, K. G. & Lindquist, S. The FLP recombinase of yeast catalyzes site-specific recombination in the Drosophila genome. *Cell* **59**, 499-509 (1989).

16 Lee, T. & Luo, L. Mosaic analysis with a repressible cell marker for studies of gene function in neuronal morphogenesis. *Neuron* **22**, 451-461 (1999).

17 Zhang, C., Williams, E. H., Guo, Y., Lum, L. & Beachy, P. A. Extensive phosphorylation of Smoothened in Hedgehog pathway activation. *Proceedings of the National Academy of Sciences of the United States of America* **101**, 17900-17907, doi:10.1073/pnas.0408093101 (2004).

18 Azpiazu, N., Lawrence, P. A., Vincent, J. P. & Frasch, M. Segmentation and specification of the Drosophila mesoderm. *Genes Dev* **10**, 3183-3194 (1996).
19 Johnson, R. L., Milenkovic, L. & Scott, M. P. In vivo functions of the patched protein: requirement of the C terminus for target gene inactivation but not Hedgehog sequestration. *Mol Cell* **6**, 467-478 (2000).
