# Supplementary material for: Hedgehog mediated degradation of Ihog adhesion proteins modulates cell segregation in Drosophila wing imaginal discs
Source: Nat Commun. 2017 Nov 2;8:1275. doi: 10.1038/s41467-017-01364-z (PMC5668237; doi:10.1038/s41467-017-01364-z)
Supplement: Supplementary file 2 — Description of Additional Supplementary Files [file 41467_2017_1364_MOESM2_ESM.docx]

**Description of Additional Supplementary Files**

File Name: Supplementary Movie 1

Description: Ihog expression in wild-type wing discs.Confocal microscope Z-stack images from the apical to the basal surface of the columnar epithelium of a wing disc immunostained for Ihog (green) and Ptc (red). Images from the same disc were presented in three ways to show Ihog and Ptc either separately (videos 1 and 2) or together (video 3). Note that representative focal planes from these videos were also shown in Supplementary Figure 4.

File Name: Supplementary Movie 2

Description: Ptc expression in wild-type wing discs. Confocal microscope Z-stack images from the apical to the basal surface of the columnar epithelium of a wing disc immunostained for Ihog (green) and Ptc (red). Images from the same disc were presented in three ways to show Ihog and Ptc either separately (videos 1 and 2) or together (video 3). Note that representative focal planes from these videos were also shown in Supplementary Figure 4.

File Name: Supplementary Movie 3

Description: Ihog and Ptc expression in wild-type wing discs. Confocal microscope Z-stack images from the apical to the basal surface of the columnar epithelium of a wing disc immunostained for Ihog (green) and Ptc (red). Images from the same disc were presented in three ways to show Ihog and Ptc either separately (videos 1 and 2) or together (video 3). Note that representative focal planes from these videos were also shown in Supplementary Figure 4.

File Name: Supplementary Movie 4

Description: Ihog expression in the wing discs from *boi* mutant larvae. Confocal microscope Z-stack images from the apical to the basal surface of the columnar epithelium of a wing disc from a *boi* mutant larva immunostained for Ihog (green) and Ptc (red). Images from the same disc were presented in three ways to show Ihog and Ptc either separately (video 4 and 5) or together (video 6). Note that representative focal planes from these videos were also shown in Supplementary Figure 5.

File Name: Supplementary Movie 5

Description: Ptc expression in the wing discs from *boi* mutant larvae. Confocal microscope Z-stack images from the apical to the basal surface of the columnar epithelium of a wing disc from a *boi* mutant larva immunostained for Ihog (green) and Ptc (red). Images from the same disc were presented in three ways to show Ihog and Ptc either separately (video 4 and 5) or together (video 6). Note that representative focal planes from these videos were also shown in Supplementary Figure 5.

File Name: Supplementary Movie 6

Description: Ihog and Ptc expression in the wing discs from *boi* mutant larvae. Confocal microscope Z-stack images from the apical to the basal surface of the columnar epithelium of a wing disc from a *boi* mutant larva immunostained for Ihog (green) and Ptc (red). Images from the same disc were presented in three ways to show Ihog and Ptc either separately (video 4 and 5) or together (video 6). Note that representative focal planes from these videos were also shown in Supplementary Figure 5.
